# Grouping Hypotheses and an Integrated Approach to Testing and Assessment of Nanomaterials Following Oral Ingestion

**DOI:** 10.3390/nano11102623

**Published:** 2021-10-07

**Authors:** Luisana Di Cristo, Agnes G. Oomen, Susan Dekkers, Colin Moore, Walter Rocchia, Fiona Murphy, Helinor J. Johnston, Gemma Janer, Andrea Haase, Vicki Stone, Stefania Sabella

**Affiliations:** 1 Nanoregulatory Platform, Drug Discovery and Development Department, Istituto Italiano Di Tecnologia, 16163 Genova, Italy; luisana.dicristo@iit.it (L.D.C.); colin.moore6@mail.dcu.ie (C.M.); 2 National Institute for Public Health and the Environment (RIVM), 3720 Bilthoven, The Netherlands; agnes.oomen@rivm.nl (A.G.O.); susan.dekkers@tno.nl (S.D.); 3 Computational Modelling of Nanoscale and Biophysical Systems—CONCEPT Lab, Istituto Italiano Di Tecnologia, 16163 Genova, Italy; walter.rocchia@iit.it; 4 Nano Safety Research Group, School of Engineering and Physical Sciences, Heriot-Watt University, Edinburgh EH14 4AS, UK; f.murphy@hw.ac.uk (F.M.); h.Johnston@hw.ac.uk (H.J.J.); v.stone@hw.ac.uk (V.S.); 5 LEITAT Technological Center, 08005 Barcelona, Spain; gemmajaner@yahoo.es; 6 Department of Chemical and Product Safety, German Federal Institute for Risk Assessment (BfR), 10589 Berlin, Germany; Andrea.Haase@bfr.bund.de

**Keywords:** ingested nanomaterials, integrated approach to testing and assessment, grouping, dissolution, durability, biopersistence

## Abstract

The risk assessment of ingested nanomaterials (NMs) is an important issue. Here we present nine integrated approaches to testing and assessment (IATAs) to group ingested NMs following predefined hypotheses. The IATAs are structured as decision trees and tiered testing strategies for each decision node to support a grouping decision. Implications (e.g., regulatory or precautionary) per group are indicated. IATAs integrate information on durability and biopersistence (dissolution kinetics) to specific hazard endpoints, e.g., inflammation and genotoxicity, which are possibly indicative of toxicity. Based on IATAs, groups of similar nanoforms (NFs) of a NM can be formed, such as very slow dissolving, highly biopersistent and systemically toxic NFs. Reference NMs (ZnO, SiO_2_ and TiO_2_) along with related NFs are applied as case studies to testing the oral IATAs. Results based on the Tier 1 level suggest a hierarchy of biodurability and biopersistence of TiO_2_ > SiO_2_ > ZnO, and are confirmed by in vivo data (Tier 3 level). Interestingly, our analysis suggests that TiO_2_ and SiO_2_ NFs are able to induce both local and systemic toxicity along with microbiota dysbiosis and can be grouped according to the tested fate and hazard descriptors. This supports that the decision nodes of the oral IATAs are suitable for classification and assessment of the toxicity of NFs.

## 1. Introduction

Nanomaterials (NMs) are being increasingly exploited by many industrial sectors, finding applications in diverse products, including food additives (to enhance texture, flavor, color, nutritional quality), food contact materials (FCM) (e.g., to passively or actively improve food and packaging stability), cosmetics and therapeutics [1,2]. Accordingly, NMs can be ingested directly (e.g., from food, water, toys and cosmetics) or indirectly (e.g., inhaled NMs are cleared via the mucociliary escalator and swallowed into the oro-gastrointestinal tract, OGI) [3,4,5,6]. There are reports of potential human, animal and environmental health impacts associated with some NMs [7], including ingested NMs. For instance, the European Food Safety Agency (EFSA) has recently updated the evaluation of the safety of the food additive titanium dioxide, E171 concluding that genotoxicity concerns cannot be excluded after consumption of TiO_2_ particles, and therefore doubts as to the levels of daily intake consumption are raised [8]. Moreover, the research community, producers and consumer associations are increasingly suggesting to policy makers the use of scientifically based evidence in order to make regulation-oriented decisions for the safe use of nanomaterials in food.

Thus, in order to provide useful strategies to streamline NM risk assessment, the EFSA, along with other agencies worldwide, recommends the use of mechanistic hazard information linked to the physical–chemical (PC) characteristics of potentially edible NMs to inform use of specific hazard endpoints [9]. For instance, if a NM undergoes complete dissolution, this results in generation of ions or molecules, and therefore toxicity can be assessed by conventional risk assessment [9,10]. In contrast, when the NM does not dissolve completely, both ions and particles may contribute to NM toxicity. EFSA defines incomplete dissolution as when 12% of the initial mass of the digested material is found in nanosized forms more than 30 min after intestinal digestion [9]. More focused testing is required to assess the toxicity of NMs which do not completely dissolve, using conditions which simulate a real exposure scenario, such as a rodent oral repeated exposure study [9,11]. Information on a number of the PCs of NMs is always needed for characterization and identification. There are some other properties, such as dissolution rate in relevant physiological media and reactivity, that may inform the impact of the NMs on living organisms [12,13,14,15], and so there is a need to focus on such measurements to gain the most useful information to support risk decision making and safe-by-design (SbD) strategies. Moreover, as a specific NM can exist in many different nanoforms (NFs) which vary in their PC characteristics such as size, shape and surface coating [16], this can influence their hazard levels. As such, the development of grouping and read-across strategies can be supportive for EU chemical legislation [17]. To this regard, collaborative efforts are established by the scientific community to advance the safety assessment of emerging NMs, suggesting different action strategies. For instance, recently, the Adverse Outcome Pathway (AOP) framework has been proposed as a supporting tool for the effective development of alternative toxicity testing strategies for NMs [18]. The implementation of the SbD approach into the industrial development of NMs has also been suggested to focus the NM design stage on NM applications and uses in a regulatory framework [19].

Within this context, the EU project, GRACIOUS, provides a framework to assist in the grouping of NFs in order to streamline hazard assessment and to support read across [20]. The framework aims at making testing more efficient, quicker and ethical by decreasing the reliance placed on animal testing [20]. Read across is a technique used for predicting endpoint specific information of a target substance (i.e., one for which data are lacking) by using data available from another similar substance (the source material) [10,21,22,23]. According to the Organization for Economic Cooperation and Development (OECD) and the European Chemicals Agency (ECHA), the rationale underpinning the grouping of NFs requires a hypothesis to be generated which defines the similarity of group members [10,22,23]. With this in mind, the GRACIOUS Framework has developed grouping hypotheses that link the PC characteristics (what they are) of NFs with information on their toxicokinetics in the human body or fate and behavior in the environment (where they go) and their hazard (what they do) [20]. Each grouping hypothesis is accompanied by a tailored, integrated approach to testing and assessment (IATA). IATAs are decision trees which have been designed to support the targeted evidence gathering and generation needed to identify whether a grouping hypothesis can be accepted or rejected, and therefore make a grouping decision. Each IATA guides the user through a series of decision nodes (DNs), where each DN asks for specific information on a relevant grouping criterion (such as PC parameters, fate and hazard biomarkers). Each DN of the IATA has a Tiered Testing Strategy (TTS) that guides the user to identify existing data, followed by the filling of data gaps using the most relevant assays/methods available. Implications of groupings are indicated so that the user can better decide on whether or not to embark on a grouping exercise. Such implications have been identified in regulatory compliance (including read across), a more efficient hazard testing, SbD processes and precautionary measures to reduce exposure [20].

The aim of this paper is to advance the grouping and read across of ingested NFs [24]. This was achieved by the formulation of grouping hypotheses and the design of specific IATAs with tailored TTSs.

## 2. Results

### 2.1. Generation of Oral Ingestion Hypotheses (H-O)

A structured analysis of existing information gathered via peer reviewed and grey literature was used to generate grouping hypotheses (see supporting materials for more details on how the gathering of existing data was performed) that conceptualize grouping of similar ingested NFs of a NM. A template (Table 1) was used to structure the hypotheses [25] that considers the purpose of performing grouping, as well as the life cycle/exposure, intrinsic PC properties (what they are), fate and toxicokinetics (where they go) and hazard (what they do) of NFs to formulate a grouping hypothesis [20,26]. This template allows hypotheses to be applied for specific purposes (e.g., SbD, regulatory, precautionary measures) which are linked to specific implications (e.g., SbD modification, inclusion in regulatory dossier to support read across). Grouping substantiating the oral hypotheses in terms of each of these elements is briefly reported in the following sections. In addition, the information on the current knowledge on toxicity of ingested NFs is outlined in Table 2.

#### 2.1.1. Linking Purpose to Implications for Grouping

The hypotheses can be used for grouping related to regulatory or precautionary measures or SbD purposes. The implications of forming a group (i.e., accepting the grouping hypothesis) may depend on the purpose of grouping. For example, for precautionary measures or SbD, the inclusion of NFs within the group of instantaneous dissolution results in loss of nano-specific properties and, therefore, provides sufficient justification to recommend following a conventional risk assessment for ionic or molecular forms of the same substance [9,10]. In the case of quick, gradual or very slow dissolution, the inclusion of NFs within either group provides a sufficient basis for recommending measures to limit exposure or accept the potential toxicity of the NF *a priori* [9].

If the grouping hypotheses are being used for regulatory purposes, the inclusion within the group of NFs which undergo instantaneous dissolution suggests the need to read across to non-NFs, with the soluble form proposed to be the most suitable [9]. In the case of quick, gradual and very slow dissolution, a stepwise approach should be adopted for hazard identification and characterization to avoid unnecessary testing. A quantitative similarity assessment should be performed to confirm that group members are sufficiently similar to perform read across [9,10].

#### 2.1.2. Lifecycle/Exposure via Oral Ingestion

NMs can be ingested intentionally (e.g., via food) or be unintentionally and indirectly ingested (e.g., leaching from food packaging into food or via clearance of inhaled particles) [3,4,5,6]. As a consequence of their increasing utilization in consumer products, NM consumption is expected for the entire consumer lifetime [3,49]. Accordingly, chronic rather than acute toxic effects on humans should be considered when conducting hazard studies by applying repeated exposure studies [50].

Metal oxide NMs, including silver, zinc, silicon, iron and titanium, are the major materials used in cosmetics (e.g., sunscreens and toothpastes), in food and in food contact materials (FCM) [50]. With respect to food, NMs are constituent parts of a variety of products such the packaging (ZnO), storage life sensors and food additives (Fe_2_O_3,_ SiO_2,_ TiO_2_) and juice clarifiers (Ag, TiO_2_) [50]. Adult consumption of NFs has been estimated to be in the order of 0.7–6.7 mg [51], 1.8 mg [52] or 2.82–4.78 μg [53] per kg of body weight (bw)/day for titanium dioxide (TiO_2_, E171), silicon dioxide (SiO_2_, E551) and silver oxide (AgO, E174), respectively. Because E171 is no longer considered safe by the EFSA [8], recently, the European Commission decided to phase out this food additive in Europe. By contrast, no daily intake consumption has been measured for NMs like zinc oxide, ZnO and iron oxide, Fe_2_O_3_, because their ionic counterpart represents a nutritional component of foods [49]. However, quantification of such NMs associated with unintentional ingestion is challenging due to poor information on product life cycles [50].

#### 2.1.3. What They Are

Intrinsic PC properties linked to fate, toxicokinetics and hazard following human exposure via inhalation, oral or dermal routes are mainly based on particle size, surface charge, specific organic coatings, aggregation and shape [54]. Size, shape, composition and surface coatings are PC properties requested in the basic information step of the GRACIOUS Framework and provide part of the information used to trigger specific hypotheses. An explanation of the role of these PC characteristics in determining where they go and what they do following NM ingestion is provided below.

#### 2.1.4. Where They Go

Consideration of where they go can be limited to where they go in the OGI tract, where they go in the body and what happens to them inside cells.

##### Where They Go: OGI Tract

With respect to the OGI tract, measurement of dissolution kinetics and of the size of NMs after the interactions between NMs and OGI simulant juices may indicate a biotransformation that is predictive of NF biopersistence in the body. For example, gradual or very slow dissolving NMs are likely to be more biopersistent [12,55]. In addition, size analysis of NFs is important in order to assess if materials are present as NMs in the OGI tract, and to what extent. Indeed, according to EFSA [56], when ≤10% of particles (number-based) in a suspension are smaller than 500 nm using a screening assay approach, this material does not require nanospecific considerations for its risk assessment, and so conventional risk assessment approaches can be applied. In contrast, if particles smaller than 500 nm are found to contribute >10% in the suspension, the applicant should show, by more thorough analysis, that ≥90% of the number of particles is greater than 250 nm. Otherwise, additional nanospecific information relative to size and the possible hazard linking needs to be provided. To this regard, the use of at least two independent techniques is needed to assess the nanoscale size in water or simple media, with one technique being electron microscopy that is conducted in association with bulk-based techniques such as Centrifugal Liquid Sedimentation (CLS) or Particle Tracking analysis (PTA) [56]. Solubility and degradation rate (dissolution) of the pristine material can be useful predictors of behaviour in the gut, although hazard effects of transformed NMs are often not considered [56]. A lack of consideration of interactions of NMs with OGI simulant juices may reduce the predictivity potential of the hazard assays. Accordingly, acellular dissolution tests which employ simulant OGI juices are therefore considered a useful approach to assess potential dissolution, which is critical in determining the bio-accessible/bioavailable fractions, which vary after biotransformation [9,57]. In addition, the surface coating of a particle can influence NM chemical stability in the OGI tract as well as mucosal and cellular penetration. For instance, it may impact dissolution, and can thereby be a predictor of accumulation or clearance in vivo [58]. For example, derivatising CeO_2_ NMs surfaces with ionisable or hydrophilic groups greatly improves solubility in water and, consequently, reduces biopersistence in vivo due to improved clearance [59]. Studies on dissolution of Ag NMs with polyvinylpyrrolidone (PVP) or citrate as capping agents have demonstrated that this may modulate the dissolution kinetics together with the aggregation of NMs. This latter effect can also impact cellular uptake efficiency [60,61]. Silane passivation of iron oxide NMs has been demonstrated to reduce dissolution in acidic lysosomal simulating conditions [55] suggesting that such coatings could contribute to biopersistance. Surface charge is also important, with net neutral or positively charged NFs easily penetrating mucus [62].

##### Where They Go: NM Translocation and Cellular Uptake

The majority of ingested NMs appear to transit through the OGI tract without being absorbed [3,59,63,64]. Indeed, following oral gavage of rats, CeO_2_ NMs demonstrated near 100% excretion via faeces [59], while AgO NMs exhibited ca. 60–90% excretion following oral gavage of rats [64]. However, for those that do enter the circulation, in vivo kinetics of particles following intestinal absorption are required. Upon diffusion in the gut and penetration of mucus, uptake requires consideration of the initial contact with enterocytes and cellular trafficking [3]. Absorption efficiency and bioavailability of particles are highly dependent on the material type used. Kim and co-authors measured the bioavailability of ZnO, TiO_2_ and SiO_2_ NMs reporting that a much higher quantity of ZnO NMs entered the bloodstream compared to the other particles, and that the effect was independent of particle size [65]. Following uptake by enterocytes, NMs can translocate to the blood stream and distribute to the liver, spleen, blood and bone marrow [66]. However, particles such as ZnO NMs appear to be absorbed and distributed to organs (liver and kidneys) in the form of zinc ions rather than in particulate form, suggesting a toxic potential of zinc ions when administered in large doses [67]. On the contrary, TiO_2_ and SiO_2_ NMs have been found mainly as particles in the main target organ, the liver [68,69]. Within cells, lysosomal (pH 4.5) dissolution of particles may impact the cell response either due to release of constituent ions or molecules. For example, metal-containing NMs accumulate in lysosomes and release their constituent ions with different dissolution rates based on many PC factors (coating, size, etc.). The released ions affect cell viability only when the ion is inherently toxic [14]. Cationic polymeric NMs can induce toxicity as a consequence of the proton sponge effect, where unsaturated amines on the material surface are capable of sequestering protons, keeping the lysosomal pump going and leading to the retention of one Cl^−^ anion and one water molecule for each proton that enters the lysosome. This process causes lysosomal swelling and rupture, leading to particle deposition in the cytoplasm [70,71,72]. Biopersistent NFs can cause autophagy and lysosomal dysfunctions resulting in toxicological consequences. Indeed, NFs can perturb the lysosome degradation of intracellular pathogens, damaged organelles and proteins, by way of the autophagy (macroautophagy), thus altering the equilibrium of these two pathways [73].

#### 2.1.5. What They Do

For NMs which enter the body via oral ingestion, a well-established hazard paradigm, as there is in the lung for fibre shaped NMs, does not exist [25,74]. However, although there are conflicting results, to date, increasing scientific evidence shows that ingested NMs could pose adverse effects [3,75]. The reported outcomes are summarized in Table 2. Local toxicity due to inflammation and genotoxic effects has been reported for ingested food grade TiO_2_ NMs (E171) in vivo [27,29,30,31,35]. For example, in rats treated for 100 days with 10 mg/kg/bw, induction of preneoplastic lesions in the colon and low-grade inflammation of the intestinal mucosa was evidenced. Particle accumulation and systemic detrimental effects, after oral exposure, in the liver and the kidneys (inflammation and/or genotoxicity) are also reported for TiO_2_, ZnO, SiO_2_, Fe_2_O_3_, CuO and Ag NMs [27,28,30,33,37,38,39,40,41,46,47,48,76], using both rat and mouse animal models. Ingested NFs can also affect the immune system responses. Indeed, Tassinari et al. reported that repeated exposure to SiO_2_ NMs (at concentrations of 2, 5, 10, 20 and 50 mg/kg/bw for 90 days by oral gavage) induced higher levels of total IgG antibodies in serum, whereas male rats were more prone to blood cell reduction [38]. Injury of cardiac function triggered by inflammation was observed after daily oral administration of TiO_2_ NMs (0, 2, 10, 50 mg/kg/bw) for 90 days in rats [32]. Numbers of reports have also reported the toxicity of ZnO NM through different mechanisms of action and a dependence upon different factors, such as concentrations, time of exposure and size of the particles [76]. Overall, the presented studies indicate that ingestion of these NMs may cause inflammation and oxidative stress in the intestine and can cause toxicity in secondary target sites (e.g., the liver).

By interacting with the gut microbiome, NMs may potentially influence the microbiota functionalities (i.e., food metabolism, intestinal integrity, systemic immune response, etc.) [3]. There is evidence that TiO_2_ NMs could induce dysfunction of gut microbiota in rats treated with dosages within the daily intake [28,34,35] The indirect pathway of oxidative stress and inflammatory response, probably due to stimulation of *lipopolysaccharides* (*LPS*) primarily produced by gut microbiota, seem to play an important role in the toxicity of ingested TiO_2_ NMs [28,34,35]. Similar evidence is also demonstrated for Ag NMs and for SiO_2_ NMs, where colon inflammation is reported because of gut microbiota alterations upon treatment of mice with 2.5 mg/kg bw of SiO_2_ NMs and 20 mL/kg/dose of Ag NMs for 7 days by oral gavage [36]. Ingestion of 26 mg/kg/bw of ZnO NMs for 30 days by oral gavage can alter the gut microbiome community of mice, but also substantially disturbs the metabolic profiles leading to neurobehavioral impairments via the microbiota–gut–brain axis [42]. However, so far limited studies exist on the interplay between NMs and the human microbiota [77]. Overall, these results suggest that gut microbiota dysbiosis induced by NMs could be the reason for local and systemic toxicity effects on the intestine and secondary target organs (i.e., brain), respectively.

This information was used to formulate the grouping hypotheses outlined in Table 1.

### 2.2. General Description of IATAs for Ingested NFs

**Figure 1:**
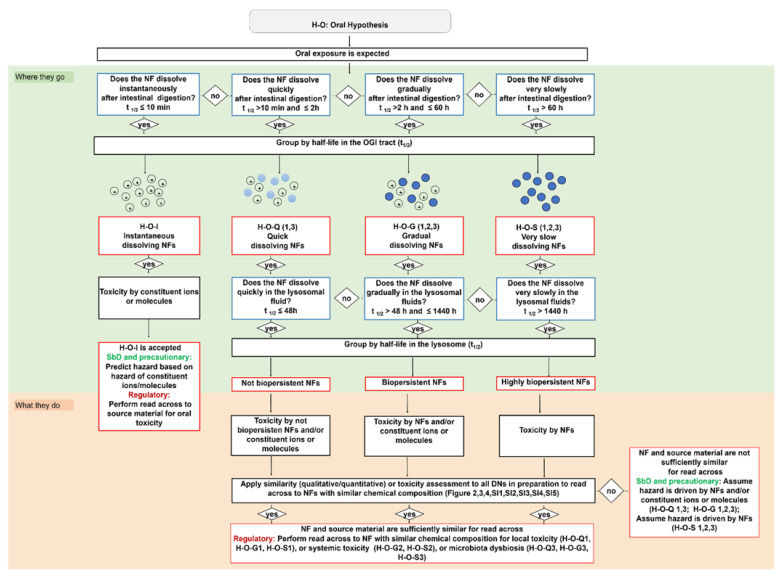
Oral IATAs are structured as decision trees that outline what information is needed to accept or reject the grouping of a target NF following ingestion. Information on dissolution kinetics is requested by the dissolution DNs (cut-offs expressed as half-lives and measured in the OGI and lysosomal simulant fluids are based on experimentally derived data) (blue boxes). Four groups of NFs (instantaneous, quick, gradual and very slow dissolving NFs) are identified in the OGI tract, which are associated with different biotransformed nano and/or molecular species (red boxes); their biopersistence is predicted by the lysosomal dissolution and further groups are generated, such as not biopersistent, biopersistent and highly biopersistent NFs (red boxes). Accordingly, the hazard can be driven by the NFs and/or constituent ions or molecules or by the NFs only. The oral IATAs may allow application of a similar toxicity assessment (vs. the source material) to define specific hazard-based groups of NFs (local or systemic toxicity or alteration of microbiota functionalities) (black boxes). Finally, a summary of how the oral IATAs differently target the user decisions depending on the grouping implications is shown (SbD/Precautionary or Regulatory). Full details for each IATA are reported in Figure 1, Figure 2 and Figure 3.

**Figure 2:**
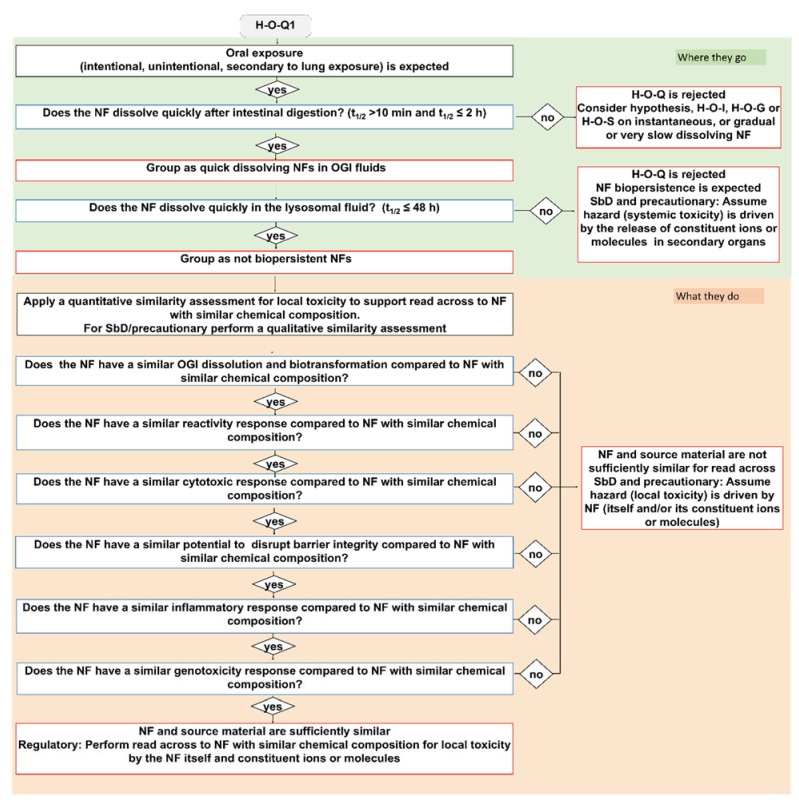
The IATA which addresses the oral ingestion hypothesis H-O-Q1: Following oral exposure, both NFs and constituent ions or molecules may contribute to local inflammation in the OGI tract, but there is no concern for NF accumulation. Blue bordered boxes are decision nodes, red bordered boxes are hypothesis conclusions, black bordered boxes are considerations.

**Figure 3:**
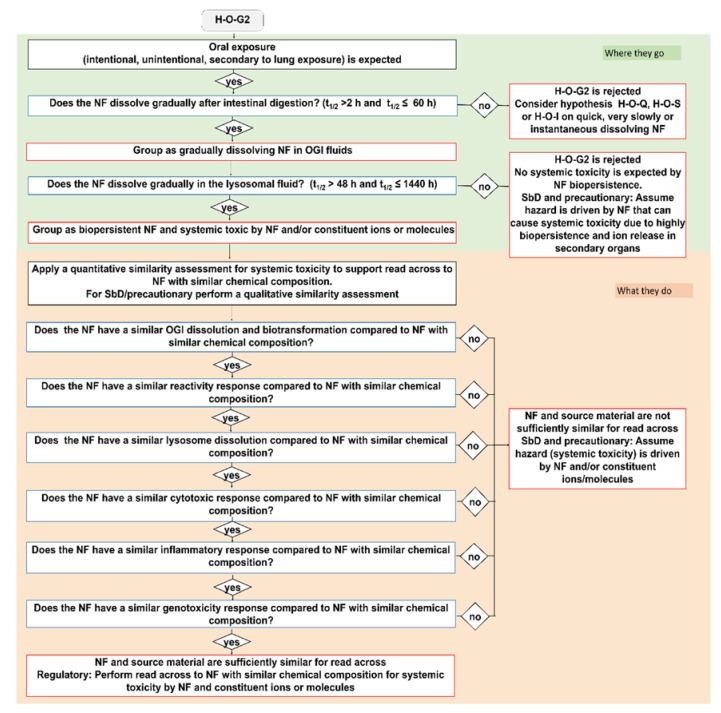
The IATA which addresses the oral ingestion hypothesis H-O-G2: Following oral exposure, both NFs and constituent ions or molecules may translocate to secondary target organs and may lead to systemic toxicity in secondary organs due to biopersistency. Blue bordered boxes are decision nodes, red bordered boxes are hypothesis conclusions, black bordered boxes are considerations.

**Figure 4:**
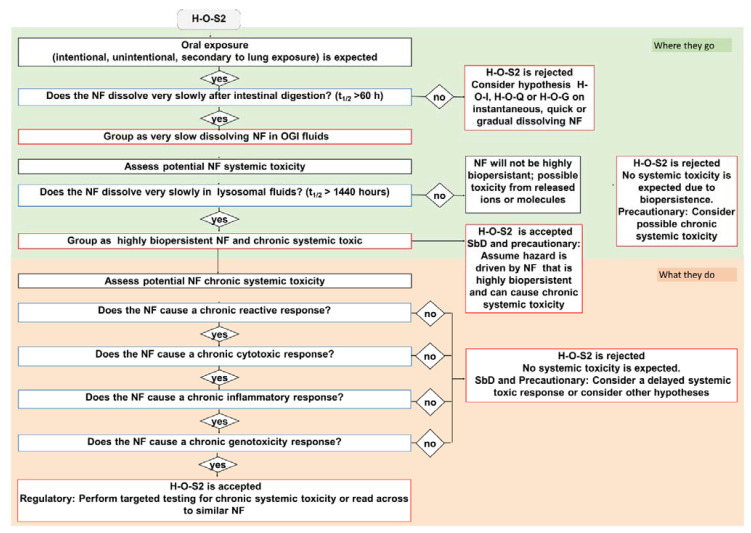
The IATA which addresses the oral ingestion hypothesis H-O-S2: Following oral exposure, NFs will maintain nanospecific activity that may drive translocation across the intestinal wall, subsequent biopersistence in the body and systemic toxicity in secondary organs. Blue bordered boxes are decision nodes, red bordered boxes are hypothesis conclusions, black bordered boxes are considerations.

Here, we describe the development of the oral IATA that has been tailored to identify the information requirements needed to justify (or reject) a grouping of a target NF. Figure 1 summarizes the key aspects common to all IATAs, focusing on the DNs related to dissolution kinetics in the OGI and lysosome, whereas the individual IATAs that show all DNs for each grouping hypothesis, including the hazard descriptors, are presented in Figure 2, Figure 3 and Figure 4 and Figures S1–S5.

Figure 1 shows that the oral IATAs start with a DN, which poses the question ‘*Does the NF dissolve instantaneously/quickly/gradually/very slowly after intestinal digestion?*’ and allows for the measurement of NF dissolution kinetics in OGI simulant fluids to assess the contribution of ions vs. particles and link this to the likelihood of biopersistence of nanospecific properties (i.e., the retention of the nanoscale size). The dissolution kinetics in the OGI tract are measured according to ISO/TR 19057 and expressed as half-life (t_1/2_) (Figure 1). By addressing this DN, the NFs are first grouped on dissolution kinetics, then hazard is considered (see Table 3 and the single IATAs). By means of three pragmatic cut-offs, the DN identifies four different groups of NFs, namely instantaneous, quick, gradual or very slow dissolving NFs. In line with the EFSA Guidance, the inclusion criterion for instantaneous dissolving NFs corresponds to a t_1/2_ ≤ 10 min [9], indicating that the NF is unlikely to persist for long enough to cross the gastrointestinal mucus layer to reach the enterocytes. The oral IATAs additionally introduce 2 h and 60 h as second and third cut-off values, to define the boundaries for quick (t_1/2_ > 10 min and ≤2 h), gradual (t_1/2_ > 2 h and ≤60 h) or slow dissolving NFs (t_1/2_ > 60 h). These pragmatic cut-offs are experimentally derived and considered physiologically relevant to reflect, by means of the dissolution kinetics values, whether constituent ions or molecules, particles or both contribute to toxicity and allow for grouping NFs according to their persistence in the OGI tract (Figure 1). For instance, a quick dissolution may indicate the potential presence of a small proportion of NFs with respect to ions, which are likely to dominate the interactions with biological components (e.g., biomacromolecules of the OGI tract or intestinal enterocytes). However, if adsorbed as particles upon crossing the mucus layer, they are likely to dissolve rapidly to constituent ions or molecules; thus, accumulation of particles in the intestine and in secondary target sites is not envisaged for this group. As the t_1/2_ increases, a greater proportion of the ingested NF is expected to persist in particulate form, which may be more likely to be adsorbed and accumulate in the body over a relatively long time [78,79]. The oral IATAs for these groups (H-O-Q 1,3; H-O-G 1,2,3; H-O-S2) therefore present an additional DN ‘*Does the NF dissolve quickly/gradually/very slowly in the lysosomal fluid?*’, which requires measurement of the dissolution kinetics in artificial lysosomal fluid to predict the potential of NFs to accumulate in secondary organs and to identify if the NFs may exert systemic toxicity [14,80]. For lysosomal dissolution, durability is assessed in simulant lysosomal fluid at the pH 4.5 to mimic the phagolysosomal fluid (PSF) of macrophages, the cells responsible for particle clearance from tissues [81]. A lower limit of t_1/2_ < 48 h indicates the timeframe in which NFs can be considered fully dissolved when inside cells [82]. When the lysosome dissolution exceeds t_1/2_ ≥ 1440 h, this value defines highly biopersistent NFs [83]. The very long timeframe between the lower and upper limit indicates the possible co-existence of NFs and constituent ions or molecules, which could pose systemic effects due to both accumulation and the release of toxic ions or molecules. In summary, the inclusion criteria for biopersistent NFs are established as t_1/2_ > 48 h and t_1/2_ ≤ 1440 h, while for highly biopersistent NFs the cut-off is a t_1/2_ > 1440 h. Accordingly, it allows for grouping different NFs based on their potential to accumulate in the lysosomes as not biopersistent, biopersistent and highly biopersistent (Figure 1). Quantification methods for the dissolution DNs (in OGI and lysosomal fluids) are reported in the TTS (Section 2.3).

Next, the IATAs further split and consider the toxicity induced by the NFs and/or constituent ions (H-O-Q1,3; H-O-G1,2,3), or by the NFs only (H-O-S1,2,3), and elaborate toxicological descriptors (i.e., reactivity, inflammation, genotoxicity, cytotoxicity and barrier impairment) accounting for local toxicity (H-O-Q1; H-O-G1; H-O-S1), (Figure 2, Figures S2 and S4) for microbiota dysbiosis (H-O-Q3,H-O-G3 and H-O-S3) (Figures S1, S3 and S5) or for systemic toxicity (H-O-G2 and H-O-S2) (Figure 3 and Figure 4).

Finally, Figure 1 summarizes how the oral IATAs target the user decisions differently depending on the purpose for grouping (e.g., precautionary/SbD or regulatory). For the quick, gradual and very slow dissolving groups, a similarity assessment (either qualitative or quantitative) applied to all the IATA DNs, which helps the user to assess whether a target NF is similar to a source material, allows grouping (toxicokinetic and hazard driven groups are presented in Table 3) and leads to the assumption that the target NF will induce similar toxicity compared to the source material. Purpose-driven decisions and working principles of the single oral IATAs are highlighted in the following sections.

#### 2.2.1. Instantaneous Dissolving NFs (H-O-I)

NFs can be grouped as instantaneously dissolving if they have a t _1/2_ ≤ 10 min. As suggested in H-O-I, if a NF is instantaneously dissolving under conditions relevant for the human OGI physiology, generation of a non-nanomaterial form is expected (Figure 1, column on the left). The IATA for H-O-I does not include any other DNs but drives the user decision depending on the purpose of grouping. (i) If the purpose is generation of a dossier to comply with relevant regulations, then application of read across to non-nanomaterial forms with the soluble constituent ionic or molecular forms is proposed to be the most suitable; (ii) if precautionary measures or SbD are requested, no nano-specific risk evaluation is needed (Figure 1, the first column reports the complete IATA for H-O-I).

#### 2.2.2. Quick Dissolving NFs (H-O-Q 1, 3)

**Figure 5:**
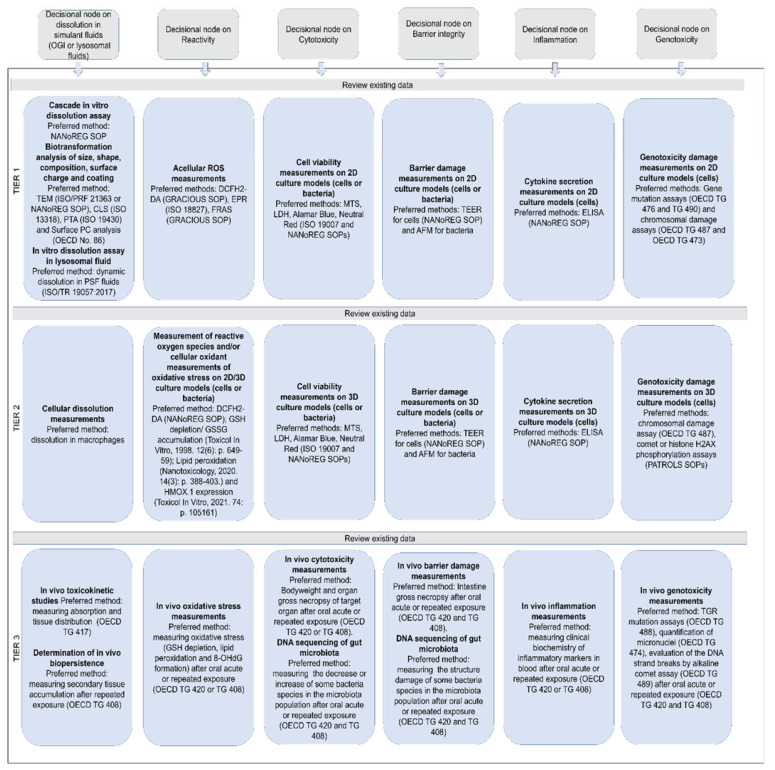
Tiered testing developed for each DN of the IATAs for hypotheses H-O-Q, H-O-G and H-O-S. The TTS defines the methods required to assess each DN of the oral IATA. Each tier starts with a recommendation to review the existing data for an effective plan of new data to generate. Progression to upper tiers will be persuaded if higher level of confidence is required. The Tier 1 indicates simple in vitro acellular assays to predict NF accumulation and durability, plus simple assays to address the biological hazards by using 2D cellular models or single strain bacterial cultures. Tier 2 indicates targeted in vitro biological testing assays by means of advanced cellular models (co-cultures and 3D models) and multi-strain biofilms or in vitro microbiome models isolated from healthy individuals. Tier 3 suggests in vivo testing assays.

The quick dissolving grouping hypothesis considers NFs that exhibit a t_1/2_ > 10 min but ≤2 h in simulant OGI juices and that do not have the potential to accumulate, as they have a t_1/2_ ≤ 48 h in lysosomal simulant fluid (Figure 1). As summarized in Table 3 and stated in the hypothesis text (Table 1), the H-O-Q hypothesis describes two hazard groups of NFs: not biopersistent and locally toxic (H-O-Q1) or not biopersistent but inducing microbiota dysfunction (H-O-Q3). As an example, the H-O-Q1 IATA is reported in Figure 2. Local toxicity is assessed by the descriptors: surface coating, reactivity, inflammation, genotoxicity, cytotoxicity and barrier impairment. The testing strategy for these parameters is tiered for the grouping purpose so they can be differently analysed accordingly: for SbD/precautionary based grouping, a qualitative similarity assessment may be sufficient to assume, based on expert judgment, that the hazard (local toxicity) is driven by NFs which do not accumulate (itself and/or its constituent ions or molecules). For example, if data do not show any differences between the tested NFs for a specific DN (i.e., through statistical analysis) the user can conclude that the NFs are similar. On the contrary, if the grouping is made to comply with chemical regulation, where read across is expected, the H-O-Q1 IATA provides guidance for a quantitative similarity assessment (using mathematically derived limits) [84] by identifying NFs with similar chemical composition (black box of Figure 1 and Figure 2: *Apply a quantitative similarity assessment to support read across to NFs with similar chemical composition*). The accompanying TTS (Figure 5 explained in the Section 2.3 of the results) provides practical guidance on how to assess the target NF versus the source material. A source material is crucial to derive a preliminary group against which the target NF must be compared. In this case, reference materials from the Joint Research Centre (JRC) repository can serve as source materials as they are data rich benchmark materials. Therefore, a non-benchmark material is intended as a target NF, hence its similarity and membership to a group is assessed using similarity toward the benchmarks. For all the tested NFs, the TTS suggests a tiered testing (validated or standardized) which reflects the level of confidence (and analytical quality) required to substantiate the grouping hypothesis. Importantly, in line with ECHA guidelines [10], the oral IATAs indicate that the quantitative similarity assessment must be applied to all DNs in the IATA [84]. Here, if the NF behaves very similarly in comparison to the identified source across all DNs (for instance, a source material that represents a worst case compared to the target material for all DNs), the similarity assessment is considered successful and poses the conditions for performing read across following the available regulatory guidelines. The IATA which supports grouping for microbiota dysbiosis (H-O-Q3) works in a similar manner, although it uses DNs and methods focused on the analysis of alteration of microbiota as reported in the Supporting Information (Figure S1).

#### 2.2.3. Gradual Dissolving NFs (H-O-G 1, 2, 3)

For the group of gradual dissolving NFs (H-O-G), both the NF (potentially with reduced size) and the soluble form of the material co-exist. To be grouped as gradual dissolving and biopersistent, the NF must exhibit a t_1/2_ between 2 h and 60 h in the OGI simulant fluids, whereas, in simulant lysosomal fluid, they must have a t_1/2_ greater than 48 h and less than 1440 h (Figure 1). The hazard groups generated by means the H-O-G hypotheses are summarized in Table 3. Here, as a representative example, we report the H-O-G2 IATA, which specifically addresses systemic toxicity (Figure 3). Also in this case, similar to H-O-Q, a qualitative or quantitative similarity assessment could be performed depending on the purpose of the grouping to ensure the grouping of biopersistent and systemically toxic NFs. The IATAs which support grouping for local toxicity (H-O-G1) and microbiota dysbiosis (H-O-G3) are reported in the Supplementary Materials (Figures S2 and S3, respectively).

#### 2.2.4. Very Slow Dissolving NFs (H-O-S 1, 2, 3)

The very slow dissolving hypothesis considers highly biopersistent NFs that exhibit a t_1/2_ greater than 60 h in simulant OGI juices. The H-O-S hypotheses are summarized in Table 3. Here, as a representative example of H-O-S IATAs, we report H-O-S2, which specifically addresses systemic toxicity (Figure 4). To be grouped as very slow dissolving and highly biopersistent, the NFs must also exhibit a t_1/2_ greater than 1440 h in lysosomal simulant fluid. Once the OGI and lysosome dissolution DNs are accepted, the IATA allows the NFs to be grouped as inducing chronic systemic toxicity and having high biopersistence (due to the potential for long-term accumulation of nanoforms in secondary organs). The H-O-S2 is then accepted with the user either assuming *a priori* as a precaution that the NF can cause chronic toxicity in secondary organs or considering further targeted testing as per the associated TTS (Figure 5). In the case of a regulatory purpose, a read across from NFs of similar composition is suggested. If the NF presents a dissolution half-life faster than 1440 h in simulant lysosomal fluid, the user rejects the hypothesis, but stringent precautionary measures can still be undertaken. Similarly, the H-O-S IATAs split to address the local toxicity (H-O-S1) and alteration of microbiota (H-O-S3) as reported in the Supporting Information (Figures S4 and S5).

### 2.3. Decision Nodes and Their Associated Tiered Testing Strategies

The following section describes each DN of the oral IATAs and the associated TTS that include assays, methods and analytical considerations for conducting IATA testing for an effective grouping of the target NF (Figure 5). A significant focus of the research community has been made to reduce use of in vivo testing by employing alternative models, such as advanced in vitro models [85,86,87]. By the employment of the TTS, we favor the use of simple in vitro acellular/cellular assays (Tier 1) and rely on their potential to predict hazard behavior of ingested NFs when compared to results found in vivo (Tier 3). However, we acknowledge that such methods can be limited in their predictivity, and so when the purpose of grouping requires more confidence, a progression of tier level into more advanced in vitro cellular based assays (Tier 2) or in vivo tests (Tier 3) is suggested by the TTS for each DN considered (where methodologies are available). For instance, in the Tier 2 level we propose the use of more advanced in vitro cell models, such as co-culture systems or 3D models. In parallel, to make the resulting data appropriate for future harmonization or benchmarking, guidelines or methods with a high level of standardization are suggested (e.g., ISO or OECD guidelines). Where standardized methods are lacking, methods validated at project level (i.e., funded projects worldwide, ongoing or delivered data from working parties of regulatory agencies) are preferred to single laboratory-developed methods or assays.

Moreover, it is important to underline that to support grouping, each NF under investigation should be assessed using the same model set-up and conditions. The DNs and the relative TTS applied to the oral hazard endpoints should be addressed using appropriate doses and exposure regimes to reflect a realistic human exposure scenario, when possible. For instance, when direct oral exposure is expected by food grade NFs, doses mimicking daily intake values are preferred, in association with repeated exposure to simulate long-term toxicological outcomes (e.g., up to five days for the treatment of intestinal cells [88] as suggested by the PATROLS EU project). The use of physiologically relevant in vitro models to better resemble the characteristics of in vivo intestinal tissues or liver/kidney tissues (site of NF accumulation) is preferred. The use of physiologically relevant models is also suggested when testing the IATAs related to microbiota dysbiosis, although no standard operating protocols (SOPs) are currently available. Here, the use of multi-strain biofilms (dual- or multi-strain cultures and biofilm models of human commensal bacteria), or in vitro microbiome models isolated from healthy individuals is preferred over the single-species bacterial cultures, as they have a great potential for providing more relevant information on NF effects on bacterial communication through physical contacts and chemical signaling in the microbiota [77]. For Tier 3 of the TTS, OECD guidelines are suggested. However, as it is not specified in these guidelines, we suggest performing the study simulating the oral ingestion of animals by food or drinking water instead of oral gavage in order to better resemble the ingestion of NFs. The following sections describe each method suggested for each DN.

#### 2.3.1. OGI Dissolution DN (H-O-I; H-O-1, 3; H-O-G 1,2,3; H-O-S 1,2,3)

To test this DN, particular attention is given to: (i) methods that mimic experimental exposure conditions and times relevant to gastrointestinal physiology; (ii) the availability of standardized methods to measure dissolution; and (iii) the availability of standard OGI simulant juices. The TTS for dissolution kinetics includes the following Tier 1 and Tier 3 methods. No suitable Tier 2 methods currently exist for this DN.

Tier 1:

○ Measurement of dissolution property by cascade in vitro dissolution assay. This method includes the consecutive addition of simulant OGI fluids (saliva, stomach and intestine) which result in molecular composition and pH jumps, transit times and volume changes, in order to reflect the passage of food through the human OGI tract [64,89,90,91]. The measurement of dissolution rate can be obtained at different elapsed times of incubation. Inclusion of a temporal point at 30 min after the addition of the intestinal simulant juice (corresponding to the sampling time of 155 min since the beginning of the test) is preferred according to the EFSA guideline [9]. The method suggested is not currently standardised, but it has been validated as an SOP within an EU project (see method in supporting information), and it is under validation through the OECD Working Party on Manufactured Nanomaterials (WPMN) (ENV/CHEM/NANO(2019)5/ADD1) [92]. Standardized fluid compositions applicable to the cascade in vitro dissolution test are accessible from ISO documents or an EU project derived SOP (NANoREG D2.08 SOP 06; ISO/TR 19057 and DIN 19738). The dissolution unit expression is based on t_1/2_ following the calculation described in Keller et al. [93,94] and is consistent with the first-order dissolution kinetics of the ISO method [95].
○ Measurements of other properties linked to dissolution and NF accumulation which may vary during the biotransformation process in the OGI.
  ▪ *Size, composition and shape.* In line with recent EFSA considerations which put emphasis on establishing analytical criteria to predict the durability of particles based on size analysis of pristine nanomaterials [56], further characterization studies of the biotransformation in the OGI tract are suggested by the TTS in support of the dissolution kinetics. Such characterization is mainly focused on size, elemental and shape analyses. At least two techniques are proposed, one of which must be microscopy based. Specifically, transmission electron microscopy (TEM) characterization coupled with a spectroscopy technique (e.g., Energy Dispersive X-ray Analysis, EDS) is suggested for nanoform size/shape distribution and elemental analysis. Standardized and validated methods are available for a semi-quantitative description of particle number distribution (ISO 21363 or NANoREG D2.10 SOP 02). Moreover, solution- based techniques can be applied, such as the CLS (ISO 13318) and PTA (ISO 19430) techniques. Dynamic Light Scattering (DLS) is not considered suitable for such analysis as it suffers a greater perturbation from large particles in polydispersed samples.
  ▪ Surface charge and coating modifications of NFs may influence dissolution kinetics. This may impact NF dispersion stability, agglomeration state, hydrophilicity, cytotoxicity, cellular penetration and circulation time in blood stream, and also their biodistribution and clearance [58]. To this regard, it is important to define whether surfaces of NFs are modified through the use of surfactants, capping agents or attached ligands. The production process provides information on surface properties. A number of methodologies could be applied depending on the nature of the NF tested as suggested by OECD No. 86. Here, only one tier level is proposed, and the user should assess the method most suitable for their NF. However, it is worth mentioning that the OECD No.86 does not currently suggest standardized assays to characterize surface coating in the OGI or lysosome like biotransformation conditions. With the growing emergence of better performing analytical techniques for the characterization of surface properties, the TTS will be updated. A list of methods includes:
    ▪ Zeta potential analysis (DLS) in the simulant OGI fluids (NANoREG D2.10 SOP 02).
    ▪ Proton nuclear magnetic resonance spectroscopy (H-NMR).
    ▪ Fourier transform infra-red (FTIR) spectroscopy.
    ▪ High-resolution transmission electron microscopy (HR-TEM).
    ▪ Inductively coupled plasma mass spectrometry (ICP-MS).
    ▪ UV-vis spectroscopy.
    ▪ X-ray photoelectron spectroscopy (XPS).
    ▪ Thermogravimetric analysis (TGA).

Tier 3:

○ In vivo toxicokinetic studies of ingested NFs may provide quantitative information on absorption and tissue distribution of NFs. Guidance for the studies may be found on OECD TG 417, which is currently under revision in order to improve the guidance applicability on nanospecific issues. One of the main limitations, in the case of ingested NFs, is that the majority of the studies quantify the total content of corresponding ions or molecules within the considered organs or tissues (e.g., blood, urine, liver) by quantitative in bulk techniques only upon tissue mineralization (e.g., Inductively Coupled Plasma Mass Spectrometer, ICP-MS). Then, limited or only indirect information on particle durability can be extrapolated. Single molecule-based techniques (microscopy or single particle ICP) may overcome such limitations, however, most of the information generally extracted is qualitative. To this regard, recently, there have been advancements in the field with the identification by spICP-MS of TiO_2_, both in the form of constituent ions and NFs [30,96,97]. For instance, in the large intestine of mice treated for three weeks with repeated administration of the food additive, E171 (5 mg/Kg/bw), there was a significant accumulation in the large intestine of Ti^4^ cations. However, TiO_2_ particle determination showed that the number of particles detected in treated mice increased as a consequence of E171 administration, and the particle size distribution closely resembled that of the original material, suggesting a slow dissolution kinetics of the tested TiO_2_ food additive [30].

#### 2.3.2. Lysosomal Dissolution DN (H-O-Q1, 3; H-O-G2; H-O-S2)

This DN predicts the potential of NFs to accumulate in the secondary body organs. It requires a minimum Tier 1 assessment of dissolution in simulant lysosomal fluid at pH 4.5 to mimic the phagolysosomal fluid (PSF) of macrophages. Accordingly, the methods of the DN are listed below.

Tier 1:

○ For lysosomal dissolution, standardized assays that describe the use of both static and dynamic systems are available. However, testing NF dissolution in dynamic conditions is considered the preferred method as the experimental results were found consistent with data available from in vivo studies [83], thus indicating the physiological relevance of the fluid motion during the dissolution process. Standardized recipes for PSF are available from ISO/TR 19057:2017. The dissolution rate is expressed, as for the dissolution measurement in the OGI tract as t_1/2_.

Tier 2:

○ This tier examines the durability in cellular systems. Cellular models to assess durability are not yet well standardized, and so there is currently no SOP available. However, studies have shown incubation of NMs with macrophages to be at least as predictive of biodurability as acellular assays for NFs and useful to clarify the specific mechanism of particle degradation [83].

Tier 3:

○ The determination of biopersistence of NFs in vivo requires long-term in vivo assays. To look at accumulation of NFs in secondary tissues, oral repeated exposure studies are recommended (OECD TG 408).

#### 2.3.3. Reactivity DN (H-O-Q1, 3; H-O-G1, 2, 3 and H-O-S1, 2, 3)

When conducting in vitro biochemical assays (cytotoxicity, inflammation, genotoxicity), it is useful to provide an understanding of the intrinsic NF reactivity (e.g., redox potential, radical formation) that may trigger toxicity in cells [10]. A more thorough TTS for reactive oxygen species (ROS) and oxidative stress is under development within the GRACIOUS Consortium. The TTS for reactivity includes the following Tier 1, Tier 2 and Tier 3 methods.

Tier 1:

Tier 1 focuses on acellular assessment of ROS production. The use of a combination of assays for regulatory implications is recommended.

○ DCFH_2_-DA (Dichlorodihydrofluorescin diacetate) assay [98].
○ EPR (Electron Paramagnetic Resonance) assay [98,99].
○ FRAS (Ferric Reduction Ability of Serum) assay [98].

Tier 2:

Measurement of ROS and/or cellular oxidant measurements of oxidative stress are recommended as a biological indicator of NF reactivity.

○ DCFH_2_-DA (NANoREG D5.06 SOP 03) to assess the presence of ROS in cellular 2D/co-cultures/3D models (intestine, liver, kidney, etc.) following a single short term (24 h) or repeated exposure to a range of NF concentrations. This assay tends to provide a negative (no ROS) or positive (ROS identified) answer but does not seem to be sufficiently sensitive to determine values in between.
○ A variety of assays are currently under evaluation, including glutathione (GSH) antioxidant depletion and glutathione disulfide (GSSG) accumulation at short time points [100], lipid peroxidation [101] and heme oxygenase 1 (HMOX-1) expression [102]. Again, such assays could be conducted using 2D/co-cultures/3D models (intestine, liver, kidney, etc.) following a single short term (24 h) or repeated exposure to a range of NF concentrations.
○ DCFH_2_-DA (NANoREG D5.06 SOP 03) to assess ROS production in in vitro microbiota models (single strain bacterial cultures or multi-strain biofilms and in vitro microbiome models isolated from healthy individuals) following a single short term (24 h) or repeated exposure to a range of NF concentrations.

Tier 3:

In vivo oxidative stress measurements of glutathione depletion and lipid peroxidation after oral acute or repeated exposure (OECD TG 420 and TG 408) are recommended. Moreover, the measurement of 8-hydroxy-2-deoxyguanosine (8-OHdG) may be also included as it is considered a pivotal marker for measuring the effect of endogenous oxidative damage to DNA [103].

#### 2.3.4. Cytotoxicity DN (H-O-Q1, 3; H-O-G1, 2, 3 and H-O-S1, 2, 3)

The gastrointestinal tract is mechanically protected by the epithelium and a layer of mucus. An intestinal tissue injury or damage could determine a measurable change in the biological equilibrium within the OGI tract, but also particle penetration into the body [3,4]. Currently, a wide range of cytotoxicity assays are available. Here, we suggest three of the most used methods for defining NF cytotoxicity, such as the lactate dehydrogenase (LDH) assay that measures plasma membrane damage (NANoREG D2.08 SOP 07), the Alamar Blue (NANoREG D5.07 SOP 06) or MTS assays (ISO 19007) that measure both the mitochondrial enzyme function and, lastly, the neutral red assay (NANoREG D5.07 SOP 06) that measures the lysosome integrity. These assays can be conducted using a range of in vitro cell models (both cells and bacteria), which can be chosen by the user to reflect either a standardised method or a specific tissue target. The TTS for cytotoxicity includes the following Tier 1, Tier 2 and Tier 3 methods.

Tier 1:

○ Cell viability assays using 2D cellular models (intestine, liver, kidney, etc.) following a single short term (24 h) exposure to a range of NF concentrations.
○ Bacteria viability using single strain bacterial cultures following a single short term (24 h) exposure to a range of NF concentrations.

Tier 2:

○ Cell viability assays using co-cultures or 3D cellular models (intestine, liver, kidney, etc.) following repeated exposure to a range of NF concentrations.
○ Bacteria viability using multi-strain biofilms and in vitro microbiome models isolated from healthy individuals following a repeated exposure for up to five days to a range of NF concentrations.

Tier 3:

○ Bodyweight and organ gross necropsy of the target organ after oral acute or repeated exposure (OECD TG 420 and TG 408).
○ DNA sequencing of microbiota population using in vivo models after repeated exposure (e.g., from rats, mice and zebrafish) (no SOPs available) to derive alteration in the microbiota population, such as a decrease or increase in some bacteria species.

#### 2.3.5. Barrier Integrity DN (H-O-Q1, 3; H-O-G1, 3 and H-O-S1, 3)

Translocation of ingested NF through the intestinal barrier is a complex phenomenon that involves their diffusion through the mucus layer, paracellular transport through inter-epithelial tight junctions and contact with M-cells that regulate the transcytosis [3,104]. Hence, damage to the intestinal barrier needs to be considered when testing NF adverse effects. To monitor the loss of the intestinal tissue’s ability to maintain its integrity, transepithelial electrical resistance (TEER) could be a useful in vitro replacement of the in vivo histopathologic analysis that qualitatively measures the tissue damage. These assays can be conducted using a range of in vitro intestinal cell models, which can be chosen by the user to reflect a standardised method. As there are no SOPs available for testing bacterial cell wall damage, we suggest using a microscope technique, when possible. The TTS for barrier integrity includes the following Tier 1, Tier 2 and Tier 3 methods.

Tier 1:

○ TEER measurement on the monolayer of 2D cellular models (NANoREG D5.03 SOP 3) following a single short term (24 h) exposure to a range of NF concentrations.
○ Damage to the bacterial cell wall and membrane on single strain bacterial cultures by AFM imaging [105] or other microscope suitable techniques following a single short term (24 h) exposure to a range of NF concentrations.

Tier 2:

○ TEER measurement of co-cultures or 3D cellular models (NANoREG D5.03 SOP 3) following a repeated exposure to a range of NF concentrations.
○ Damage to the bacterial cell wall and membrane on multi-strain biofilms and in vitro microbiome models isolated from healthy individuals by atomic force microscopy (AFM) imaging [105] or other suitable techniques following a repeated exposure to a range of NF concentrations.

Tier 3:

○ Intestine gross necropsy after oral acute or repeated exposure (OECD TG 420 and TG 408).
○ DNA sequencing of the microbiota population using in vivo models after repeated exposure (e.g., from rats, mice and zebrafish) (no SOPs available) to derive alteration in the microbiota population, such as structure damage of some bacteria species.

#### 2.3.6. Inflammatory Response DN (H-O-Q1; H-O-G1, 2 and H-O-S1, 2)

Another important key endpoint for measuring the level of NF toxicity (local and systemic) is the induction of a pro-inflammatory response. In in vivo studies, inflammation is commonly monitored by immunohistochemical staining or by quantifying the inflammatory serum biomarkers (e.g., cytokines, C-reactive protein, etc.). The most used in vitro assay that measures the activation of a pro-inflammatory response is the use of ELISA methods to determine the amount of pro-inflammatory cytokines (e.g., IL-6, IL8 and TNF-alpha) released by cells. These assays can be conducted using a range of in vitro cell models, which can be chosen by the user to reflect either a standardised method or a specific tissue target. The TTS for inflammation includes the following Tier 1, Tier 2 and Tier 3 methods.

Tier 1:

○ Cytokine secretion measurement on supernatants collected from 2D cellular models (intestine, liver, kidney, etc.) following a single short term (24 h) exposure to a range of sub-lethal NF concentrations (NANoREG D5 06 DR SOP 06).

Tier 2:

○ Cytokine secretion measurement on supernatants collected from co-culture or 3D cellular models (intestine, liver, kidney, etc.) following a repeated exposure to a range of sub-lethal NF concentrations (NANoREG D5 06 DR SOP 06).

Tier 3:

○ Clinical biochemistry of inflammatory markers in blood after oral acute or repeated exposure (OECD TG 420 and TG 408).

#### 2.3.7. Genotoxicity Response DN (H-O-Q1; H-O-G1,2 and H-O-S1,2)

NF ingestion can also induce local and systemic genotoxicity responses; thus, in vitro genotoxicity assays should be performed on both models of intestine and secondary organs to investigate local and systemic toxicity. A detailed TTS for this DN is under development within the GRACIOUS Consortium. For Tier 1 and Tier 2 testing it is proposed that genotoxicity assays are performed according to the OECD guidelines using a range of in vitro cell models, which can be chosen by the user to reflect either a standardised method or a specific tissue target. However, moving toward Tier 2 is recommended for a better understanding of secondary DNA damage. Moreover, as suggested by OECD guidelines, when there is a positive outcome from a single in vitro study, the user should go through the Tier 3 level.

Tier 1:

○ Gene mutation assay using the Hprt and xprt genes (OECD TG 476) or the Thymidine Kinase Gene (OECD TG 490) using 2D cultures (intestine, liver, kidney, etc.) following a single short term (24 h) sub-lethal exposure to a range of NF concentrations.
○ Chromosomal damage by the quantification of micronuclei (OECD TG 487) and the identification of structural chromosomal aberrations (OECD TG 473) using 2D cultures (intestine, liver, kidney, etc.) following a single short term (24 h) sub-lethal exposure to a range of NF concentrations.

Tier 2:

○ Chromosomal damage by the quantification of micronuclei (OECD TG 487) using 3D cultures (intestine, liver, kidney, etc.) following a repeated exposure to a range of sub-lethal NF concentrations.
○ Comet or Histone H2AX phosphorylation assays (PATROLS SOPs) using 3D cultures (intestine, liver, kidney, etc.) following a repeated exposure to a range of sub-lethal NF concentrations.

Tier 3:

○ Transgenic Rodent (TGR) mutation assays (OECD TG 488) using tissues (intestine, liver, kidney, etc.) from exposed animals after oral acute or repeated exposure (OECD TG 420 and TG 408).
○ Quantification of micronuclei (OECD TG 474) in the cytoplasm of interphase cells of erythrocytes from bone marrow and/or peripheral blood cells after oral acute or repeated exposure (OECD TG 420 and TG 408).
○ Evaluation of the DNA strand breaks by alkaline comet assay using tissues (intestine, liver, kidney, etc.) from exposed animals (OECD TG 489) after oral acute or repeated exposure (OECD TG 420 and TG 408).

### 2.4. Testing the Oral IATAs: Preliminary Grouping by Dissolution in the OGI Tract

If the implications for grouping are SbD/precautionary, the oral IATAs advise that the hypothesis is tested by addressing the toxicokinetic DNs using experimentally and literature-based information:

#### 2.4.1. Does the NF Dissolve Instantaneously, Quickly, Gradually and Very Slowly after Intestinal Digestion?

**Figure 6:**
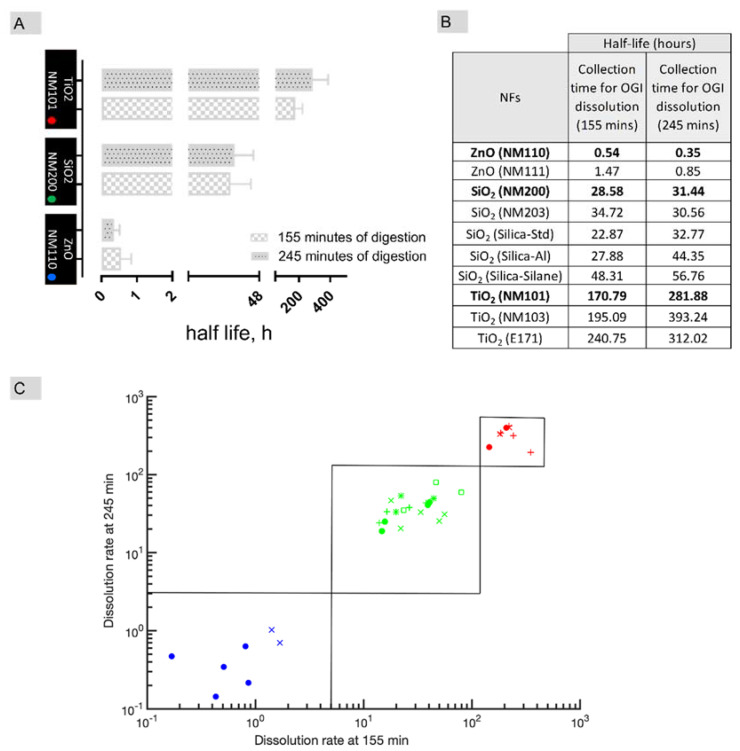
A case study to test the suitability of the oral IATAs for grouping of NFs. (**A**) Histograms report the dissolution half-life (t_1/2_) of benchmarks materials (ZnO NM110, SiO_2_ NM200 and TiO_2_ NM101) measured after 155 min and 245 min of OGI digestion. Data are expressed in hours and as mean ± standard deviation (*n* = 4). (**B**) Table reporting the average half-life values of benchmarks (in bold) and target NFs in simulated OGI fluids. Data are expressed in hours (*n* = 4). (**C**) NFs are represented on a logarithmic 2D graph having the half-life of OGI digestion estimated after 155 min and 245 min on the x- and y-axes, respectively. The points corresponding to different NFs are represented according to a colour code based on composition (Titanium-based in red, Silica-based in green and Zinc-based in blue). Closed red circles refer to NM101; red crosses refer to NM103, red plus refers to E171; Closed green circles refer to NM200; green crosses to NM203, green plus refers to Silica_sd; green asterisk refers to Silica_Al; green square refers to Silica_Silane; closed blue circles refer to NM110 and blue crosses refer to NM111. In the scatter plot, the main component of the NF is clearly the main determinant for the clustering of the points, as sketched by solid line rectangles.

To test the oral IATAs ZnO (NM110), SiO_2_ (NM200) and TiO_2_ (NM101) NMs (from the Joint Research Centre, JRC repository) were selected as relevant benchmark materials [106]. Tier 1 of TTS was then tested to measure the dissolution kinetics by the cascade in in vitro dissolution assay (refer to Supporting materials for methods). Figure 6A reports the t_1/2_ values calculated according to Keller et al. [93,94] after 155 min and 245 min of digestion (the first time corresponds to the first 30 min of incubation within intestinal simulant juice upon its addition to the mixture of saliva and stomach, and the second time to the end of the process). After 155 min of digestion, the t_1/2_ values for ZnO (NM110), SiO_2_ (NM200) and TiO_2_ (NM101) were ca. 0.5, 28.5 and 170.8 h, respectively. These values associate well with the pre-defined cut-offs of oral IATA, allowing the ZnO NF to locate to the quick dissolving group (H-O-Q), SiO_2_ to the gradual dissolving group (H-O-G) and, finally, TiO_2_ to the very slow dissolving group (H-O-S). The dissolution measurements lead to the conclusion that ZnO NFs is a quickly dissolving material, which aligns with EFSA indications using the same collection time (155 min) for dissolution measuring [9]. Moreover, when the intestinal digestion time increases (245 min), we noted that the grouping decision did not change, and similar conclusions can be drawn from the calculation of t_1/2_ values (Figure 6A,B). However, it is worth noting that there was a mild tendency for SiO_2_ and TiO_2_ NFs t_1/2_ to increase with time, thus suggesting that as the digestion process occurs in the complete digestive juice (saliva, stomach and intestine), a slowing down of the dissolution occurs for these two NFs (Figure 6A,B). In line with recent findings [107], these effects might indicate differences in chemistry driven ion solubility over time.

#### 2.4.2. Does the NF Dissolve Quickly, Gradually and Very Slowly in Lysosomal Simulant Fluid?

The dissolution in lysosomal fluid was evaluated referring to literature-based information on benchmark materials, according to the test proposed in Tier 1 of the TTS. The data indicate that among the three benchmarks, NM101 was confirmed to be the most biodurable material in lysosomal-like conditions [108] while NM110 was observed to be quick dissolving and silicon-based NFs, such as the NM200, showed a gradual dissolution behavior [109].

#### 2.4.3. Preliminary Grouping Exercise

The data generated on benchmark materials from OGI fluid dissolution were used to conduct a preliminary grouping exercise based on a similarity assessment to support inclusion of target NFs in the pre-defined group. As target NFs, we refer to NFs which lack data and which have a similar chemical composition to their benchmarks. The t_1/2_ of the target NFs was therefore compared to that obtained by the benchmarks (Figure 6B,C). The target NFs, although chemically similar to NM110, NM200 and NM101, differ in some PC properties (e.g., coating, size and crystalline structure) or in the manufacturing procedures which are reported in Table S1. A logarithmic 2D graph estimates the half-life in OGI fluid after 155 min and 245 min on the *x*- and *y*-axes, respectively, for all NFs tested (Figure 6C). In this representation, the particles quite neatly cluster around the source NFs (full dots), thus confirming membership in the pre-defined groups (H-O-Q, H-O-G, H-O-S). Interestingly, no differences in dissolution kinetics due to the impact of specific organic coatings or manufacturing processes are evidenced by the similarity analysis that may lead to different groups (Table S1 for details in the PC differences). However, for a clearer understanding of how the PC properties can establish a group of NFs, a systematic investigation by tuning each single property in a multidimensional based similarity approach, e.g., different coatings in diverse NFs with similar and constant size and chemical composition, may be beneficial to enable a specific grouping based on such properties. In the case of titanium-based materials, membership in the very slow dissolving group is also confirmed for the food grade E171, which is mostly composed of micro-sized TiO_2_ particles but with a nanosized (<100 nm) fraction less than 3.2% by mass, and so cannot be completely considered a NF [51]. This demonstrates the ability of the exercise to extend beyond NFs. However, the tested target NFs applied in this study are limited in number, and an extensive validation including more diverse datasets within more complex scenarios will be required in future studies to validate the proposed groupings via oral IATA. Indeed, there are complex systems, such as the food additive E551, which are available on the market as aggregated forms of engineered nanomaterials [52,110,111]. The selection of one or more source materials (which may be representative of one or more of the features of the aggregated systems) along with the application of quantitative approaches to assess similarity either in a pairwise or multi-component analysis [84] will be the key to enable grouping via oral IATA. The IATA suggests different levels/amounts of information and different levels of quality/standardization depending on the purpose of grouping. However, in the case of aggregated systems, the use of a good representative source material, intended as a Tier 3 data-rich benchmark material, is essential to calibrating the grouping results obtained by the lower tiers.

### 2.5. Tier 1 Data of Oral IATAs: Are They Predictive of Tier 3?

In this case study, we used the information derived from in vivo published literature (Tier 3) to understand if Tier 1 dissolution data (OGI and PSF fluids) can be used to reliably assess similarity. The Tier 3 data supported the feasibility of using the Tier 1 methods to allow acceptance of the oral hypotheses H-O-Q, H-O-G, H-O-S. The process used to come to this conclusion is briefly summarized below.

#### 2.5.1. Toxicokinetics Results from In Vivo Literature Data ì

ZnO NFs (H-O-Q)

- In rats, after single dose oral administration of commercialized ZnO NFs, zinc ions that correspond to 90% of initial administered particle mass were found to be excreted via faeces three days post exposure. Zinc ions and not zinc particles were found mainly distributed among organs such as the liver, lung and kidney [67].
- Long term administration (270 consecutive days) to mice of food replenished with commercialized ZnO-NPs showed no significant accumulation of zinc in the main tissues/organs, even though some focal-like inflammatory cells appeared to accumulate in the liver, both in the parenchyma and around the central vein [40].
- Sub-acute oral exposure to commercialized ZnO NFs (28 consecutive days) of mice reported that 60–65% of zinc in tissue (liver and kidney) is in the ionic form, and one third part, or 30–35%, is in the non-ionic form, demonstrating a fast dissolution of zinc particles during oral administration. Moreover, ZnO NFs caused an up-regulation of the hepatic pro-inflammatory cytokines, leading to the activation of acute phase response (APR) [44].
- In mice exposed to a single dose oral administration of commercialized ZnO NFs, the concentrations of Zn in the blood, liver, kidneys, spleen and lungs were significantly increased at 4 h and 12 h after ZnO NFs administration, whereas at 24 h, the accumulation of Zn could only be detected in liver and kidneys, suggesting a fast recovery of Zn levels in mice within 24 h [45].

TiO_2_ NFs (H-O-S)

- Very limited bioavailability after single oral exposure to rats is reported for realistic doses of TiO_2_ NFs (NM100, NM101, NM102, NM103 and NM104); however, there was evidence that absorption is possible in the gastrointestinal tract, as increased levels of titanium could be detected in the livers and mesenteric lymph nodes in exposed animals. Elimination was very slow (no clear differences between titanium dioxide-exposed animals and vehicle-treated controls) up to 90 days post-exposure, suggesting a potential tissue accumulation. Moreover, this process was most pronounced for the pigment-sized (NM100), and one of the nano-sized, titanium particles (NM102) [112].
- By using spICP-MS, Talamini and co-workers demonstrated that TiO_2_ E171 particles were located in the intestines of treated mice after repeated oral exposure (3 days/week for 3 weeks) [30].

SiO_2_ NFs (H-O-G)

- Liver, kidney and spleen were the target organs for silica accumulation after repeated oral exposure to realistic doses of SiO_2_ NFs (NM200 and NM203) [37,38].
- After a single oral exposure to commercialized colloidal silica NFs by rats, particles were identified by TEM analysis in their pristine form in the liver [68].
- Commercialized colloidal and food grade SiO_2_ NFs were mostly excreted by faeces after a single oral exposure to rats [68,113].

#### 2.5.2. Hazard Results from In Vivo Literature Data (Table 2)

Local Toxicity (H-O-S1)

- TiO_2_ food grade induced oxidative stress (superoxide production measurement) in the stomach and inflammation at the intestinal level [30,35], plus a disruption of the intestinal mucus barrier [35].
- Development of preneoplastic lesions occurred in the colon following chronic exposure to E171 particles [27,29,31].
- Systemic Toxicity (H-O-S2)
- Liver accumulation of titanium was also associated with an increased number and size of necro-inflammatory foci containing tissue monocytes/macrophages in E171 fed mice [30].
- Adverse systemic effects were also reported in the heart [32], where a 90-day exposure to commercialized anatase TiO_2_ NFs provoked changes in heart rate and blood pressure, with cardiac impairment detectable at the level of blood molecular markers.
- Microbiota Dysbiosis (H-O-S3)
- Food grade and commercialized nano TiO_2_ induced changes in gut microbiota, especially mucus-associated bacteria [35], a gut microbiota fluctuation with a decreased abundance of Lactobacilli in faeces [34], and a shift in gut microbiota composition in a time-dependent manner [114].

Local Toxicity (H-O-G1)

- Commercial SiO_2_ NFs can activate intestinal infection and inflammatory responses by diminishing the function of the intestinal mucus barrier [35].
- Increased pro-inflammatory cytokine levels (IL-1β, IL-6 and TNF-α) were observed in the colon of mice that ingested a commercialized SiO_2_ NF [36].

Systemic Toxicity (H-O-G2)

- A 90-day oral exposure to SiO_2_ NFs (NM203) induced enlarged sinusoids in the liver of male rats [38].
- A vacuolization of tubular epithelial cells occurred in the kidney (after 18 months of exposure via drinking water to NM200), as well as a reported inflammatory response in the livers of exposed animals. Here, the urine test detected proteinuria that the authors associate to a glomerular dysfunction [37].
- Short-term exposure (from 24 to 45 h) to SiO_2_ NFs (NM202, NM203, NM200 and NM201) did not induce DNA damage in various organs of rats, either directly or through oxidative stress, as assessed by the comet and micronucleus assays. However, the authors did not exclude that some secondary genotoxic effects following long-term exposure to SiO_2_ NFs may occur [115]. Indeed, during biomonitoring of workers involved in colloidal SiO_2_ NF production, some evidence of early, still reparable, genotoxic and oxidative effects were reported, but the authors conclude that discrimination between the effects due to NFs or other chemicals used in the NM production process are not possible, and further studies are needed [116].

Microbiota dysbiosis (H-O-G3)

- Exposure to food grade SiO_2_ NFs led to changes in gut microbiota, especially in mucus associated bacteria, decreasing some bacteria families in a dose dependent manner [35].
- Commercialized SiO_2_ NFs ingestion in mice increased microbial species richness and diversity within the intestinal tract and, in particular, an obvious increase in the genus Lactobacillus was recorded [36].

Taken together, these data demonstrated the feasibility of the oral IATAs in producing groups of NFs (which are determined by the similarity in dissolution kinetics in simulant OGI and lysosomal juices) by using the benchmark materials for which Tier 3 information was available. Interestingly, the in vivo literature data relative to Tier 3 confirm the predictivity of the dissolution information collected experimentally by Tier 1, thus suggesting a hierarchy of biodurability and persistence, which for the tested NFs is as follows: TiO_2_ NFs (H-O-S) > SiO_2_ (H-O-G) > ZnO (H-O-Q). From the toxicological data reported, it also appears that TiO_2_ and SiO_2_ NFs are able to induce local and systemic toxicity (inflammation, genotoxicity and intestinal epithelial barrier damage) (H-O-S1, 2 and H-O-G1,2) and microbiota dysbiosis (H-O-S3 and H-O-G3), though it should be noted that there is currently no consensus that these materials lead to the adverse effects and there may be differences between different NFs of the same material. Altogether, this suggests that the DNs used to develop the oral IATAs are suitable for assessing the oral toxicity by ingested NFs and the formation of groups. In conclusion, if the implications for grouping are precautionary or SbD, addressing dissolution of DNs allows acceptance of the grouping hypothesis according to the predefined oral hypotheses, H-O-Q, H-O-G and H-O-S. In the future, with the growing emergence of in vivo information in association with quantitative similarity tools [84], a robust grouping validation leading to the inclusion of more target NFs within more complex scenarios will be possible.

## 3. Discussion

The current work provides nine hypotheses that describe how ingested NFs could be grouped, along with tailored IATAs, which identify the most relevant PC, kinetic and hazard descriptors needed to support the grouping decision. The oral IATAs consist of DNs that guide the user on what information is needed to make a grouping decision for a target NF. Each DN is accompanied by a TTS, which prioritizes the application of simple in vitro assays (acellular- and cellular-based) to gather the information required for each DN in the IATA. The TTS allows performance of Tier 1 experiments that can be complemented by more advanced testing (Tier 2 and Tier 3) when an increasing confidence level is required for toxicokinetics and hazard outcomes, suggesting that not all the tiers of testing must always be performed. Indeed, by providing fate/hazard-based information, the higher tiers provide evidence of physiologically relevant similarity to underpin more effective groups, especially in the case of a regulatory purpose. As result, the oral IATAs and the groupings they may derive are driven not only by the hazard endpoints but also by the different purposes of grouping.

Central to the oral hypotheses and IATAs is the dissolution DN, a PC determinant, which, when measured in the OGI or lysosomal simulant fluids, may predict the retention of the nanoscale size of the NF in vivo, thus identifying the molecular species (NF only or nanoforms and/or constituent ions or molecules) that may potentially cause toxicity. Solubility or dissolution in water at acidic pH are suggested as simple methods to inform on the behavior of NF surface corrosion and dissolution [9,56]. However, oral dissolution is a kinetic process governed by complex equilibria, where PC parameters related to the NF itself and simulant juice can affect the entire process. The physiological relevance of the assay, for the molecular compositions selected and the exposure times, is central to obtain dissolution kinetics in conditions which, as much as possible, are close to the reality of NF ingestion. For the above reasons, to assess dissolution kinetics, the oral IATA suggests preferred in vitro methods which apply the cascade modality (the consecutive addition of simulant juices that more closely resemble the OGI environment, i.e., presence of digestive enzymes, bile salts and proteins and organic/inorganic salts whose concentration vary over the digestion time). Standard preparation recipes for the juices are available and are suggested in the TTS, as well as a cascade method that is currently under validation though OECD WPMN.

This work also uses literature-based information in association with newly generated data on dissolution to identify the cut-off boundaries needed to categorize NFs as exhibiting instantaneous, quick, gradual and very slow dissolution. The rationale underpinning the choice of the cut-off values provided for the dissolution rate was based on biologically relevant timeframes that reflect the time required for a NF to reach the intestinal epithelium and be taken up by cells. These values are therefore flexible and can be adapted if newly experimental data (following the TTSs) suggest other cut-off values are more appropriate. These dissolution outcomes are linked to different purposes in order to derive appropriate grouping decisions that are proportional and relevant to the purpose of grouping. Indeed, recognizing the purpose and context for grouping helps to streamline the NF risk assessment. For instance, to support SbD, a user may quickly assess whether an ingested NF is biopersistent or not by testing only the dissolution DNs in simulant OGI and lysosomal fluids, and therefore can easily screen a set of NFs. In this way, a grouping according to the oral hypotheses may also be used to promote the adoption of precautionary measures, especially for materials on which limited hazard data are available. On the contrary, grouping and read across for regulatory purposes may require a greater degree of scientific justifications, and therefore will require data driven evidence by quantitative similarity-based assessment between the target and an identified source NF for each of the DNs described above.

Notably, ingested NFs can be grouped by hazard specific descriptors, defining groups of NFs presenting local toxicity, systemic toxicity and microbiota dysbiosis. These descriptors were substantiated by an extensive review of the current literature of toxicity induced by ingested NFs. In particular, to the best of our knowledge, the oral IATAs, defined for the first time, fate and hazard descriptors group NFs with a clear impact on gut microbiota. Moreover, the TTS suggests, where possible, the application of relevant advanced in vitro cellular models (co-cultures or 3D cultures for intestine or secondary organs and microbial biofilms), together with standardized or validated methods to provide the basis for a more effective and robust future benchmarking in the grouping field. The implemented TTS can be amended if new protocols will become available. By this methodological approach, three benchmark materials (NM110, NM200 and NM101), for which Tier 3 information is available, have been assessed for the OGI dissolution DN, finding that they can be considered candidate source materials to perform grouping studies. Interestingly, the approach underlying the IATA and the testing strategy is general, and it also allows the testing of organic materials (nano- and non-nano NMs, such as pigments, that are case study materials within the GRACIOUS Framework). Indeed, the possibility to use a wide range of molecules accounting for minimal changes in the organic moieties will support the validation of the IATA along with the related similarity tools, possibly highlighting the strengths and the weaknesses of the strategy.

## 4. Conclusions

We have described the development of oral IATAs supporting evidence-based grouping hypotheses for ingested NFs in relation to their fate and hazard. The implementation of the IATAs in tiers of increasing specificities and complexities can support the user’s decision to accept or reject the predefined grouping hypotheses, avoiding the need for extensive testing. Indeed, the oral IATA decision trees illustrate how NF dissolution in OGI fluids (durability indication), NF dissolution in PSF fluid (biopersistence indication) and the hazard evaluation can be integrated to substantiate the grouping of ingested NFs. The grouping approach and IATAs presented here lead to grouping of NFs by dissolution. Thus, these descriptors can be used to provide justification for hazard classification depending on the purposes (regulatory and precautionary/SbD) and are able to accelerate the risk assessment of NFs. Grouping approaches are considered valid analytical tools to increase the efficiency of risk assessments for NFs. At the same time, the increasing public and legal demand for replacement of animal testing by relevant in vitro alternatives can be addressed. In summary, the IATAs provided here contribute to the use of grouping of NFs relevant to the ingestion exposure route.

## Figures and Tables

**Table 1:** (Above): GRACIOUS template for generating grouping-based hypotheses [20,25]. (Below) Human oral hypotheses (H-O-) developed for oral ingested NFs.

**Purpose of Grouping and Context**		
**Lifecycle and Exposure**		**What they are**
		**Where they go**
		**What they do**
**Implications of grouping**		
** 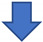 **		
**Human oral hypotheses**		
**H-O-I**	**NFs with an instantaneous dissolution: Following oral exposure, the toxicity is driven by and is therefore similar to that of the constituent ions or molecules.**	
**H-O-Q1**	**NFs with a quick dissolution: Following oral exposure, both NFs and constituent ions or molecules may contribute to local inflammation in the OGI tract, but there is no concern for NF accumulation.**	
**H-O-Q3**	**NFs with a quick dissolution: Following oral exposure, both NFs and constituent ions or molecules may drive antimicrobial impacts (e.g., reducing microbial content and diversity within the OGI tract), but there is no concern for NF accumulation.**	
**H-O-G1**	**NFs with a gradual dissolution: Following oral exposure, both NFs and constituent ions or molecules may lead to local inflammation in the OGI tract.**	
**H-O-G2**	**NFs with a gradual dissolution: Following oral exposure, both NFs and constituent ions or molecules may translocate to secondary target organs and may lead to systemic toxicity in secondary organs.**	
**H-O-G3**	**NFs with a gradual dissolution: Following oral exposure, both NFs and constituent ions or molecules may drive antimicrobial impacts, such as reducing microbial content and diversity within the OGI tract.**	
**H-O-S1**	**NFs with a very slow dissolution: Following oral exposure, NFs will maintain nanospecific activity that may lead to local inflammation within the OGI tract.**	
**H-O-S2**	**NFs with a very slow dissolution: Following oral exposure, NFs will maintain nanospecific activity that may drive translocation across the intestinal wall, subsequent biopersistence in the body and systemic toxicity in secondary organs.**	
**H-O-S3**	**NFs with a very slow dissolution: Following oral exposure, NFs will maintain nanospecific activity that may drive antimicrobial impacts, such as reducing microbial content and diversity within the OGI tract.**	

**Table 2:** Recent hazard data on the commonly ingested NMs. Only studies that consider oral exposure were selected. * doses within the NF daily intake.

Nm	Type	Model	Dose	Exposure Time	Effect		Reference
					Where they go	What they do	
TiO_2_	E171 (food additive)	Rats	10 mg/kg/bw	100 days by drinking water	Titanium was detected in the immune cells of Peyer’s patches (PP)	Preneoplastic lesion formation in the colon and induction of mucosal low-grade inflammation	[27]
TiO_2_	Anatase NM	Rats	* 2, 10, 50 mg/kg/bw	90 days by oral gavage	not assessed	Diversity of gut microbiota in rats increased in a dose-dependent manner. LPS produced by gut microbiota increased significantly; Hepatotoxicity, including clear mitochondrial swelling at highest dose	[28]
TiO_2_	E171 (food additive)	Mice	* 5 mg/kg/bw	21 days by oral gavage	not assessed	Induction of oxidative stress and immune response pathways, activated genes for DNA repair and both up- and down-regulated genes involved in development of cancer (e.g., colon cancer)	[29]
TiO_2_	E171 (food additive)	Mice	* 5 mg/kg/bw	3 weeks by dripping of the NF suspension into the mouth of mice	Accumulation of Ti in the liver and the intestine; TiO_2_ particle accumulation in large intestine	Liver and intestine inflammation; Oxidative stress in the stomach	[30]
TiO_2_	E171 (food additive)	Mice	* 5 mg/kg/bw	10 weeks by oral gavage	Some particles were internalized in colonic cells	Dysplastic alterations were observed in the distal colon	[31]
TiO_2_	Anatase	Rats	* 2, 10, 50 mg/kg/bw	90 days by oral gavage	not assessed	Impairment of cardiac function induced by inflammatory events (increase in TNF-α and IL-6 in serum)	[32]
TiO_2_	Anatase	Rats	* 2, 10, 50 mg/kg	90 days by oral gavage	Limited absorption and distribution of Ti in organs	Increase in ROS markers mostly in liver	[33]
TiO_2_	Anatase	Rats	* 1, 50 and 100 mg/kg/bw	7 and 14 days	Ti accumulated in the intestine and liver. Feces excretion	Inflammation in colonic tissue; Gut microbiota alteration	[34]
TiO_2_	Anatase Food-grade micro and nano	Mice	10, 40, and 160 mg/kg/bw (micro-TiO_2_); 10, 40, and 160 mg/kg/bw (nano-TiO_2_)	28 days by oral gavage	No significant difference in Ti content tissues compared with the control	Intestine inflammation (greatest for nano-TiO_2_); Alteration of gut microbiota	[35]
SiO_2_	Amorphous	Mice	* 2.5 mg/kg bw	7 days by oral gavage	not assessed	Increased pro-inflammatory cytokine in the colon; Alteration of gut microbiota	[36]
SiO_2_	Amorphous	Mice	* 4.8 mg/kg bw	18 months by drinking water	Si levels higher in kidneys and liver	Histological abnormalities in kidneys, Inflammatory responses in livers; perivascular amyloid accumulation in liver	[37]
SiO_2_	Amorphous	Mice	25, 160, and 300 mg/kg/bw	28 days by oral gavage	Si content in the colon was significantly higher (160 mg/kg/bw)	Intestine inflammation; Alteration of gut microbiota	[35]
SiO_2_	Amorphous	Rats	* 2, * 5, 10, 20 and 50 mg/kg/bw	90 days by oral gavage	Si accumulation in liver and in spleen (female rats)	Enlarged sinusoids in liver (male); Thyroid stimulating hormone (TSH) and creatinine levels were affected (female), higher levels of total IgG antibodies in serum (female); blood cell count reduction (male)	[38]
ZnO		Mice	300 mg/kg/bw	14 days by oral gavage	Zn ions accumulation in the liver	Elevated alanine aminotransferase (ALT) and alkaline phosphatase (ALP); Pathological lesions in the liver; Genotoxicity damage in liver and kidneys	[39]
ZnO	NM, microparticles and Zn ions	Mice	1600 mg/kg/bw	270 days by food	Excretion mainly through feces. Zn ions accumulated only in the digestive tract organs	Liver lesions induced by microparticles, but fewer by NF and Zn ions	[40]
ZnO	NM and zinc sulfate	Mice	250 mg/kg/bw	7 days by oral gavage	Elevated Zn concentrations in serum, liver, and kidney	Zinc sulfate shows more severe and acute toxicity; Zinc NFs reduce the body weight and increase serum glutamic-pyruvic transaminase activity	[41]
ZnO		Mice	26 mg/kg/bw	30 days by oral gavage	not assessed	Neurobehavioral impairment; Alteration of gut microbiota	[42]
ZnO	NM	Rats	100 mg/kg/bw	75 days by oral gavage	not assessed	Hepatic and renal damages that may subsequently cause mitochondrial dysfunctional which instigating the generation of ROS and oxidative stress	[43]
ZnO	NM	Mice	10, 30, and 300 mg/kg/bw	28 days by oral gavage	Elevated Zn concentration in liver and kidney	Severe damages in liver and kidney tissue; Hepatic proinflammatory cytokines up-regulated; increased in expression of hepatic acute phase proteins; Altered interlinked iron signaling biomarkers	[44]
ZnO	NM	Mice	1 g/kg/bw	Single dose by oral gavage	Elevated Zn concentrations in liver and kidneys	Enriched Fe level in the blood and slight increase in Liver; Decreased Fe level in the kidneys, spleen and lungs; Limited organ damages in the livers and kidneys	[45]
Fe_2_O_3_		Rats	100 and 200 mg/kg/bw	Single dose by oral gavage	Iron deposits in hepatocytes and Kupffer cells.	Inflammation in the liver	[46]
CuO		Rats	* 1 to 512 mg/kg/bw	5 days by oral gavage	not assessed	Changes in hematology parameters; Clinical chemistry markers (liver damage) histopathological alterations in bone marrow, stomach and liver	[47]
Ag NMs		Mice	20 mL/kg/dose	7 days by oral gavage	not assessed	Shifts in the intra- and inter abundance of families of gut bacteria	[36]
Ag NMs		Rats	20, 40, 60, 80 and 100 mg/kg bw	12 weeks by oral gavage	Accumulation of silver in liver, not completely eliminated from the body	Impairment of liver and kidney enzymes	[48]

**Table 3:** Hazard based groups of NFs generated by means of similarity assessment (for the case of H-O-Q and H-O-G) or toxicity assessment (for the case of H-O-S) via oral human hypotheses and related IATAs.

**H-O-Q (1,3), H-O-G (1,2,3) and H-O-S (1,2,3)**		
**Hazard descriptors** **(Reactivity, Inflammation, Genotoxicity Cytotoxicity, Barrier impairment)**		
**Group by hazard descriptors**		
**H-O-Q1** **Locally toxic NF** **but not biopersistent**	**H-O-G1** **Locally toxic NF**	**H-O-S1** **Locally and chronic toxic NF**
	**H-O-G2** **Biopersistent and systemically toxic NF**	**H-O-S2** **Highly biopersistent and chronic systemically toxic NF**
**H-O-Q3** **Inducer of microbiota dysbiosis but not biopersistent**	**H-O-G3** **Inducer of microbiota dysbiosis**	**H-OS3** **Chronic inducer of microbiota dysbiosis**

## Data Availability

The datasets used and/or analyzed during the current study are available from the corresponding author on reasonable request.

## Supplementary Materials

The following are available online at https://www.mdpi.com/article/10.3390/nano11102623/s1, Figure S1: The IATA which addresses the quick dissolving oral ingestion hypothesis related to microbiome dysbiosis (H-O-Q3). Figure S2: The IATA which addresses the gradual dissolving oral ingestion hypothesis related to local toxicity (H-O-G1). Figure S3: The IATA which addresses the gradual dissolving oral ingestion hypothesis related to microbiota dysbiosis (H-O-G3). Figure S4: The IATA which addresses the very slow dissolving oral ingestion hypothesis related to local toxicity (H-O-S1). Figure S5: The IATA which addresses the very slow dissolving oral ingestion hypothesis related to microbiota dysbiosis (H-O-S3). Table S1: PCs characteristics of the tested NFs.
