# Supplementary material for: Grouping Hypotheses and an Integrated Approach to Testing and Assessment of Nanomaterials Following Oral Ingestion
Source: Nanomaterials (Basel). 2021 Oct 7;11(10):2623. doi: 10.3390/nano11102623 (PMC8541163; doi:10.3390/nano11102623)
Supplement: Supplementary file 1 [file nanomaterials-11-02623-s001.zip › nanomaterials-1377667-supplementary.pdf]

Supplementary Information

Grouping Hypotheses and an Integrated Approach to Testing and Assessment of Nanomaterials Following Oral Ingestion

Luisana Di Cristo [1], Agnes G. Oomen [2], Susan Dekkers [2], Colin Moore [1], Walter Rocchia [3], Fiona Murphy [4], Helinor J. Johnston [4], Gemma Janer [5], Andrea Haase [6], Vicki Stone [4] and Stefania Sabella [1,*]

[1] Nanoregulatory Platform, Drug Discovery and Development Department, Istituto Italiano Di Tecnologia, Genova16163, Italy; luisana.dicristo@iit.it (L.D.C.); colin.moore6@mail.dcu.ie (C.M.)

[2] National Institute for Public Health and the Environment (RIVM), Bilthoven 3720, The Netherlands;
agnes.oomen@rivm.nl (A.G.O.); susan.dekkers@tno.nl (S.D.)

[3] Istituto Italiano Di Tecnologia, Computational Modelling of Nanoscale and Biophysical Systems—CONCEPT Lab, Genova 16163, Italy; walter.rocchia@iit.it

[4] Nano Safety Research Group, School of Engineering and Physical Sciences, Heriot-Watt University,
Edinburgh EH14 4AS, UK; f.murphy@hw.ac.uk (F.M.); h.Johnston@hw.ac.uk (H.J.J.); v.stone@hw.ac.uk (V.S.)

[5] LEITAT Technological Center, Barcelona 08005, Spain; gemmajaner@yahoo.es

[6] Department of Chemical and Product Safety, German Federal Institute for Risk Assessment (BfR), Berlin 10589, Germany; Andrea.Haase@bfr.bund.de

* Correspondence: stefania.sabella@iit.it

Gathering of existing *in vivo* data. An overview on available *in vivo* data used to justify grouping of ingested NFs was collected by the analysis of public scientific literature using online resources as PubMed and Google scholar database with the following keywords: oral exposure to nanomaterials/nanoparticles, ingested nanomaterials/nanoparticles with a focus on NFs accumulation, clearance and toxicity (local, systemic and microbiota alteration). Dates of research cover the period from January to February 2021.

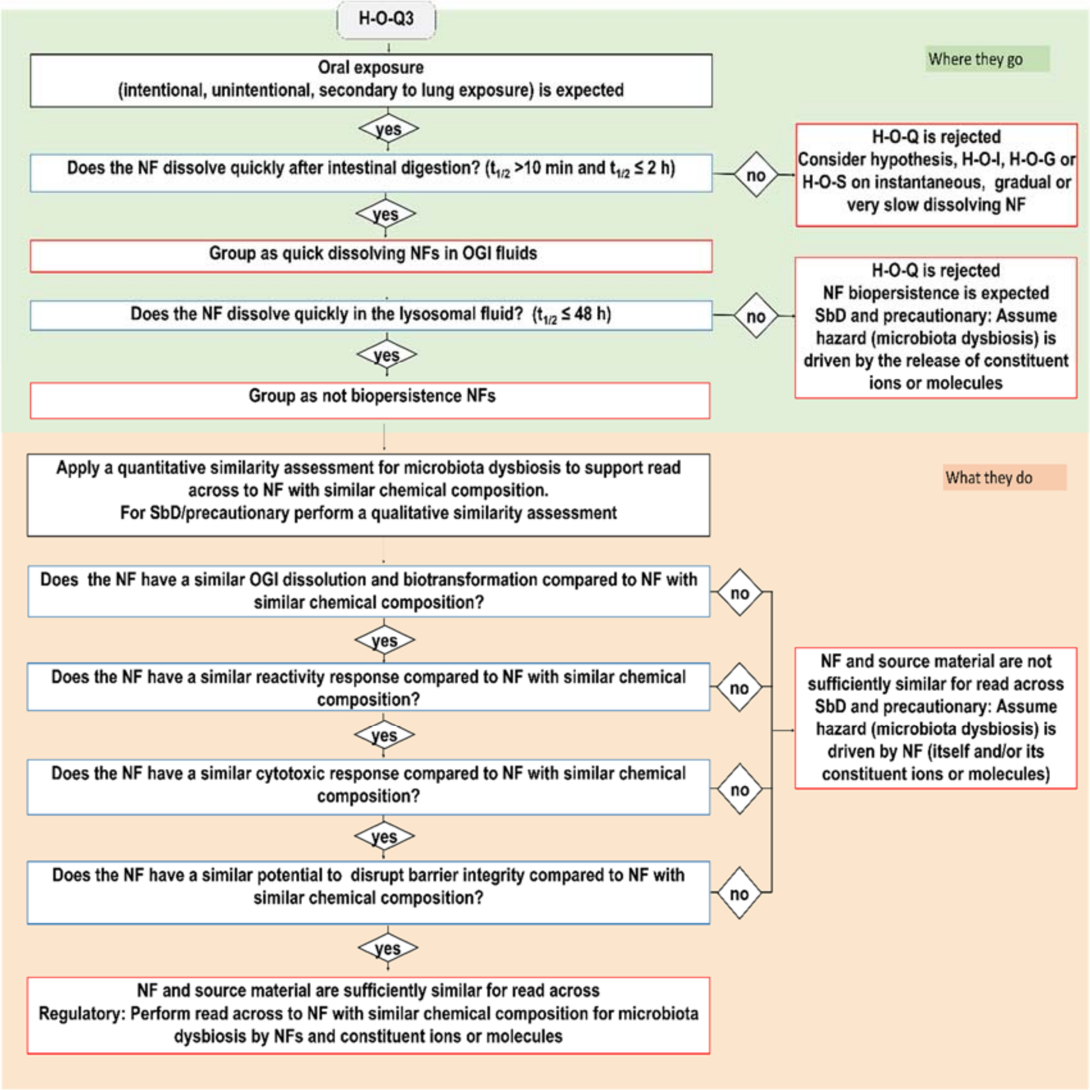

Figure S1. The IATA which addresses the 'quick dissolving' oral ingestion hypothesis related to microbiome dysbiosis (H-O-Q3). Following oral exposure both NFs and constituent ions or molecules may drive antimicrobial impacts (e.g., reducing microbial content and diversity within the OGI tract), but there is no concern for NF accumulation. Blue bordered boxes are decisional nodes, red bordered boxes are hypothesis conclusions, black bordered boxes are considerations.

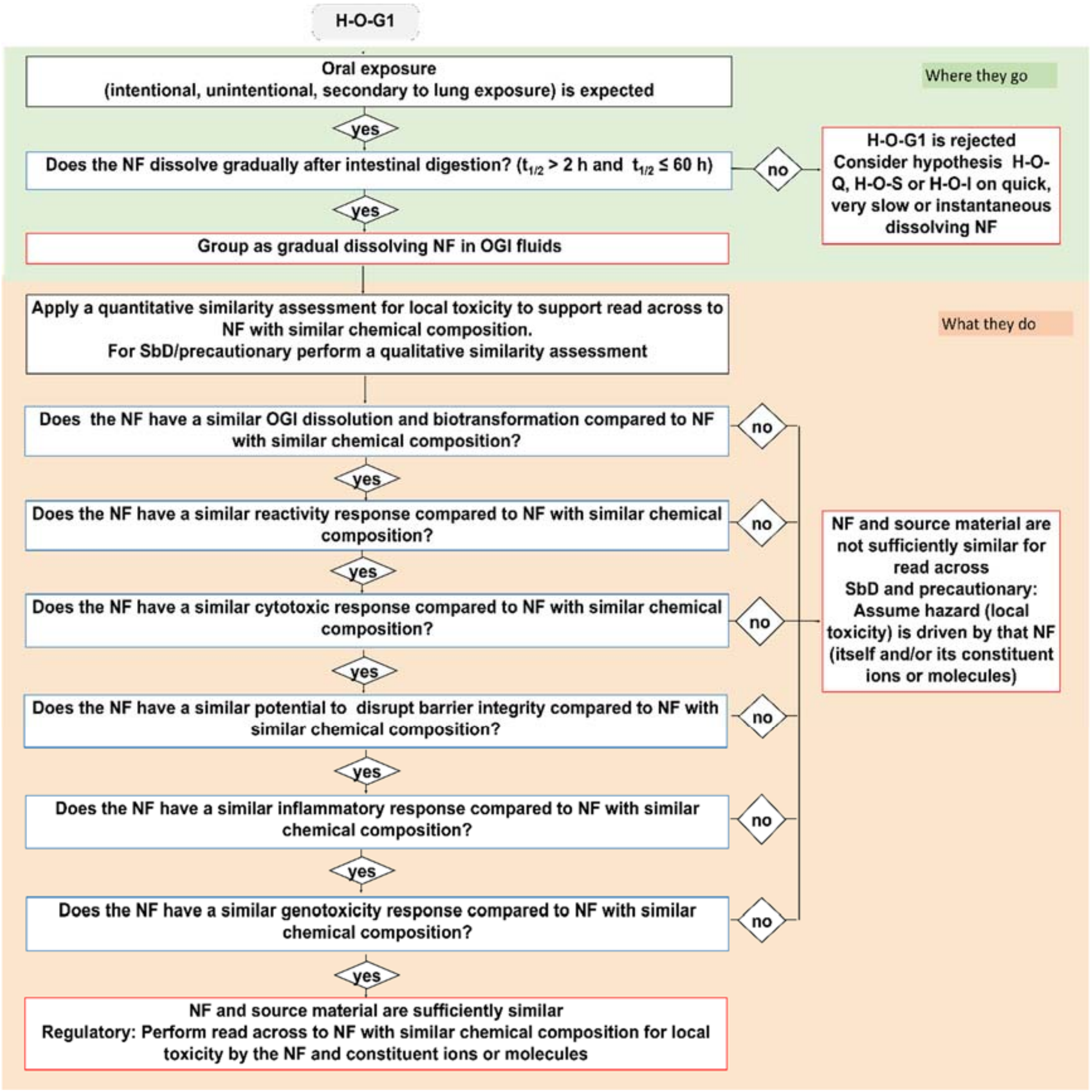

Figure S2. The IATA which addresses the 'gradual dissolving' oral ingestion hypothesis related to local toxicity (H-O-G1). Following oral exposure both NFs and constituent ions or molecules may lead to local inflammation in the OGI tract. Blue bordered boxes are decisional nodes, red bordered boxes are hypothesis conclusions, black bordered boxes are considerations.

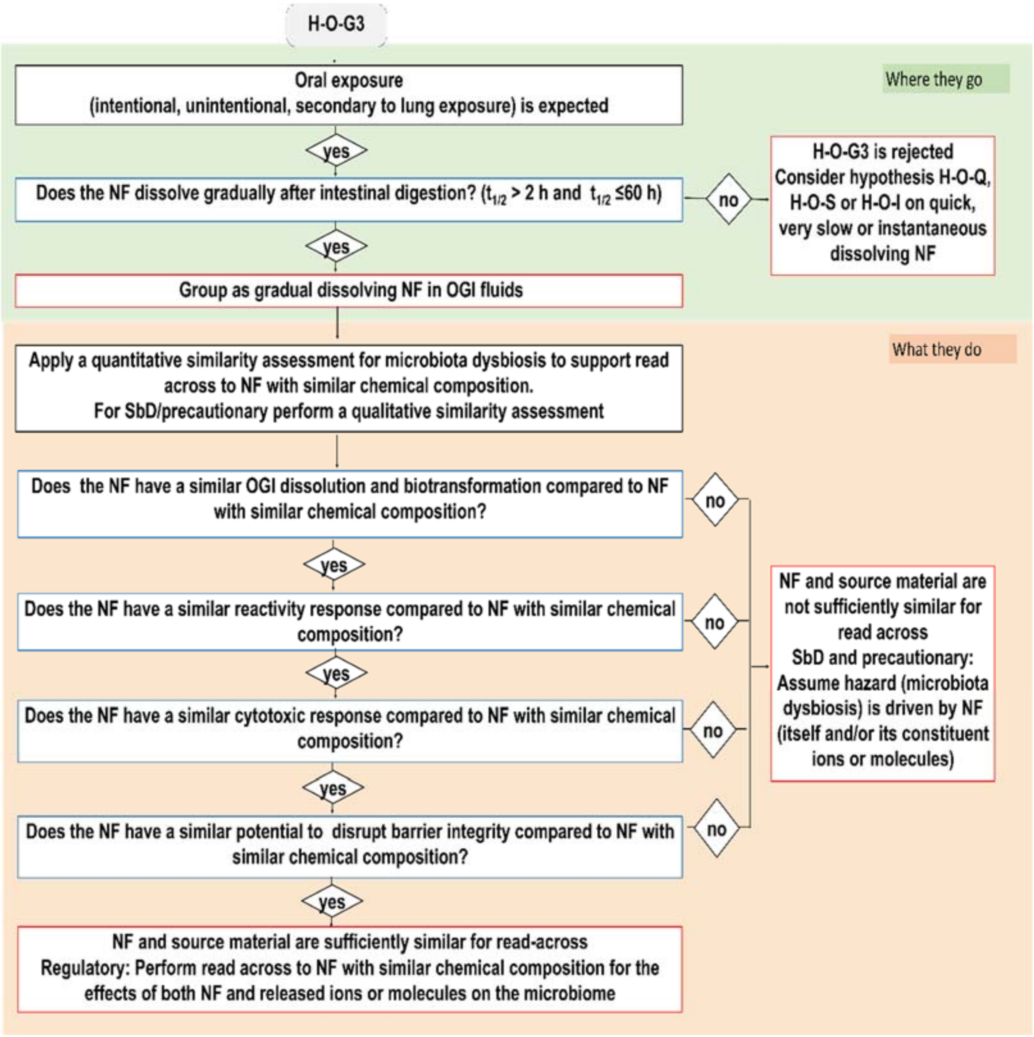

Figure S3. The IATA which addresses the 'gradual dissolving' oral ingestion hypothesis related to microbiota dysbiosis (H-O-G3). Following oral exposure both NFs and constituent ions or molecules may drive antimicrobial impacts, such as reducing microbial content and diversity within the OGI tract. Blue bordered boxes are decisional nodes, red bordered boxes are hypothesis conclusions, black bordered boxes are considerations.

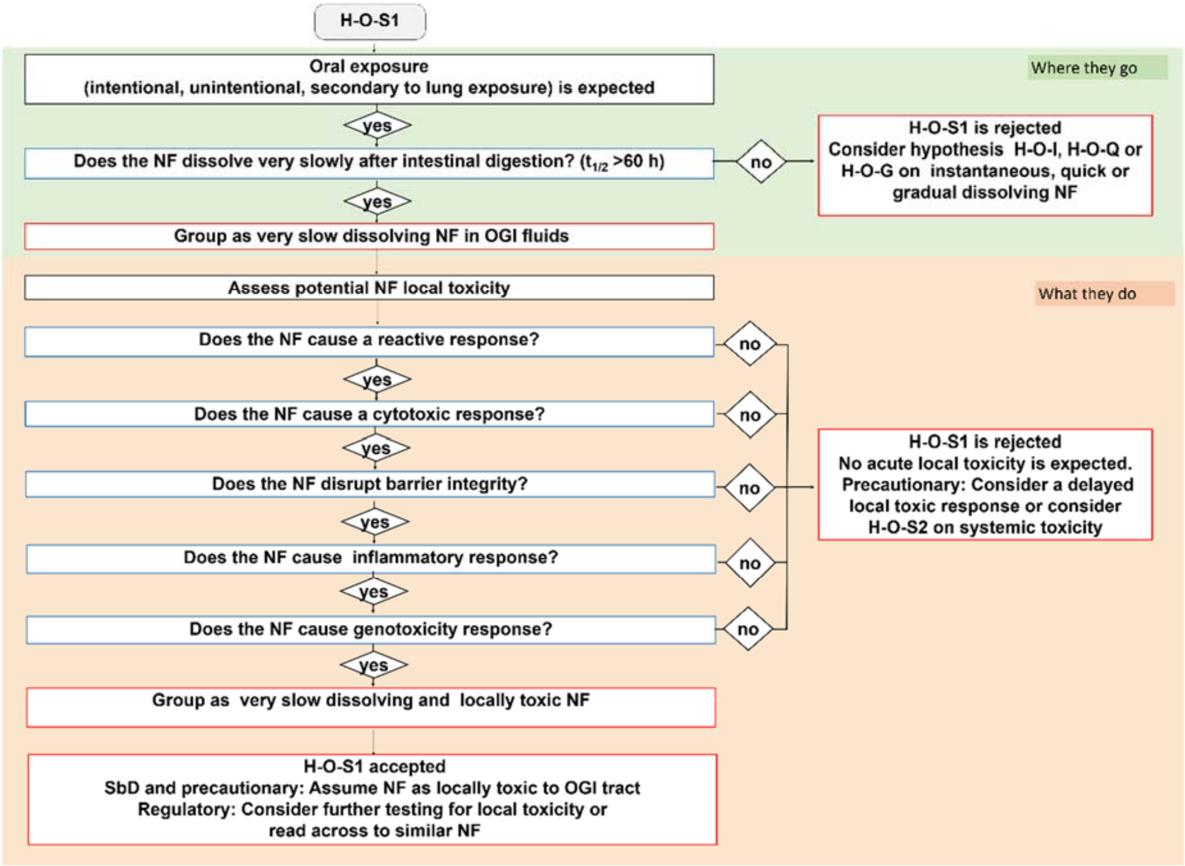

Figure S4. The IATA which addresses the 'very slow dissolving' oral ingestion hypothesis related to local toxicity (H-O-S1). Following oral exposure NFs will maintain nanospecific activity that may lead to local inflammation within the OGI tract. Blue bordered boxes are decisional nodes, red bordered boxes are hypothesis conclusions, black bordered boxes are considerations.

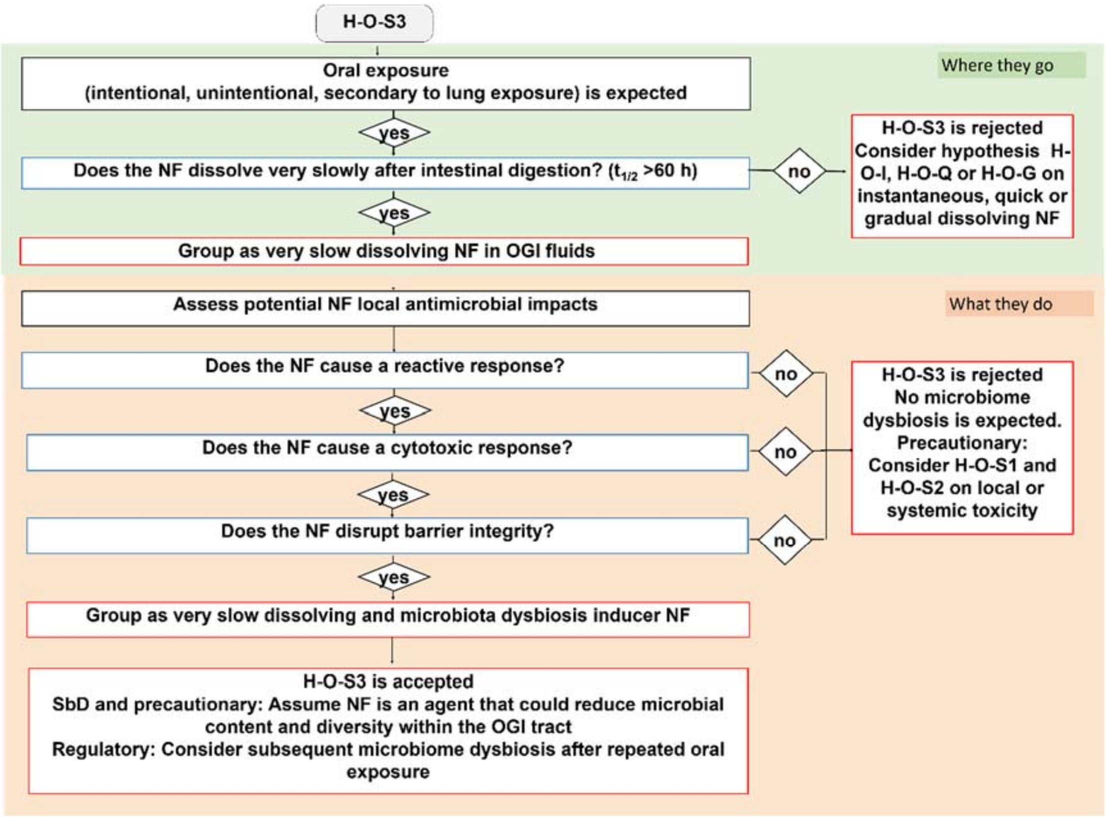

Figure S5. The IATA which addresses the 'very slow dissolving' oral ingestion hypothesis related to microbiota dysbiosis (H-O-S3). Following oral exposure NFs will maintain nanospecific activity that will drive antimicrobial impacts, such as reducing microbial content and diversity within the OGI tract. Blue bordered boxes are decisional nodes, red bordered boxes are hypothesis conclusions, black bordered boxes are considerations.

Nanoforms. NM101, NM103, NM110, NM111, NM200 and NM203 were obtained from the JRC Nanomaterials Repository (Ispra, Varese, Italy). The E171, Silica_Std, Silica_Al and Silica_Silane were commercially materials provided within the GRACIOUS project. Food grade non-nano TiO_2, E171, was obtained from Venator (Germany), whereas Silica NFs were obtained from Nouryon (Sweden). The table below provides a list of the main physico chemical characteristics of the selected NF [1-5].

Table S1. PCs characteristics of the tested NFs

NF	Type of material	Size (nm)	Specific surface area (BET, m2/g)	Coating
NM110	Zinc Oxide	158	12,4	uncoated
NM111	Zinc Oxide	152	15	triethoxycaprylsilane
NM101	Titanium Dioxide (anatase)	5,5	316,07	uncoated
NM103	Titanium Dioxide (rutile)	24	50,83	Aluminum
E171	Titanium Dioxide (anatase)	204	15	uncoated
NM200	Silicon Dioxide (precipitated)	25	189	uncoated
NM203	Silicon Dioxide (thermal)	29	203	uncoated
Silica-Std	Silicon Dioxide (colloidal)	11	209	uncoated
Silica-Al	Silicon Dioxide (colloidal)	13	175,5	Na-aluminate
Silica-Silane	Silicon Dioxide (colloidal)	11	215,6	glycerol-propyl moieties from alkyl-tri-alkoxysilanes

Cascade in vitro dissolution assay. In order to simulate the digestion process a modified version of the *in vitro* dissolution test described by our recent works was followed [6,7]. Here the overall volume of the process was scaled down two-fold to increase its practicality. NFs was dispersed in MilliQ water at the concentration of 2.56 mg/mL. The stock suspension was then sonicated using "Sonopuls Ultrasonic Homogenizers HD2200" equipped with 3mm probe. The power applied during the sonication was 30% amplitude for 5 minutes. The suspension was then diluted in MilliQ water at the working concentrations, of 50 μg/mL (ZnO and TiO_2 NFs) and 1000 μg/mL (SiO2 NFs). Juices (saliva, stomach, duodenum and bile) were prepared the day of the experiment and were pre-heated to 37°C before starting the digestion process. To begin, 0.500 ml of NFs at different concentrations was transferred into a falcon tube. The digestion process started when 3 mL of saliva juice was added to the NF suspension. After 5 minutes at 37°C (shaking at 80 RPM), 6mL of stomach juice was added and the falcon tube were then incubated for other 120 minutes at 37°C (shaking at 80 RPM). At the end, 6 ml of duodenal fluid, 3 ml of bile salts and 1 mL of 84.7 g/L sodium bicarbonate were added before further 30 and 120 minutes of incubation at 37°C (shaking at 80 RPM) (corresponding to the cumulative sampling time of 155 min and 245 min, respectively).

Ultrafiltration (UF) and Inductively coupled plasma - optical emission spectrometry (ICP-OES). Ultrafiltration of digested samples was performed using Amicon Ultra 15 mL centrifugal 3K filters according to the manufacturer's instructions. The flow-throughs containing free ions only was then processed by ICP-OES. Samples (TiO_2 and ZnO) were dissolved in Aq. Regia (Hydrochloric acid, HCl and Nitric acid, HNO3, ratio 3:1) at a final concentration of 10% v/v with MilliQ water and then analyzed by ICP-OES (Agilent 720/730 spectrometer). Silica samples manipulation was carried out in order to minimize silicon background (silicon release from equipment) using ultrapure reagents and disposable polypropylene tubes. Samples were then dissolved in hydrofluoric acid (HF) at a final concentration of 10% v/v with MilliQ water. ICP calibration standards were used to construct a multipoint standard curve.

Dissolution rate calculation. Dissolution rate of NFs after the digestion process was derived following the calculation described in Keller et al [1] and it is consistent with the first-order dissolution kinetic of ISO 19057:2017 [8]. In brief, the total ion mass dissolved at time t [Mion (t)] obtained by ICP analysis in intestine (after 155 and 245 minutes) was used to derive the dissolution k rate as follows:

$$K_{dis} = \frac{M_{ion}(t)}{SA\ (t)} / \Delta t \quad (1)$$

Δt is the sampling interval time over the entire digestive process. Two sampling times were selected: 155 minutes and 245 minutes of NF incubation, which corresponds, the first, to the 30 minutes elapsed from the addition of the intestinal simulant juice to the mixture of saliva and stomach, and the second time is the end of process. SA (t) is the specific surface area at time t and is approximated as:

$$SA(t) = BET(t_0) * (M_0 - M_{ion}\ (t)) \quad (2)$$

SA is obtained by multiplying the Brunauer–Emmett–Teller (BET) value at time 0 (t0) to the M_0-Mion(t), where M_0 is the total ion mass of the NF at time 0, supposing a 100% of dissolution (diluted with the respect of compartment dilution factor in which NFs undergo in the process (i.e., 1:39 for the intestine). The inclusion of the BET value is important as it links the k_{dis} to the surface area of the NFs. By the equation (2), the conventional expression unit of k is $ng/cm^2/h$. Dissolution k rate can be converted to dissolution half-life $t_{1/2}$, expressed in hours, by:

$$t_{1/2} = \frac{\ln(2)}{BET * K_{dis}} \quad (3)$$

Statistical Analysis. Data are expressed as mean values ± standard deviation. Differences have been considered significant for p values < 0.05. Statistical analysis was conducted using GraphPad Prism 8.4 (GraphPad Software Inc., La Jolla, CA, USA). An Unpair Student's t test was performed.

References

(1) Keller, J.G., W. Peijnenburg, K. Werle, R. Landsiedel, and W. Wohlleben, *Understanding Dissolution Rates via Continuous Flow Systems with Physiologically Relevant Metal Ion Saturation in Lysosome.* Nanomaterials (Basel), 2020. **10**(2).

(2) Keller, J.G., M. Persson, P. Müller, L. Ma-Hock, K. Werle, J. Arts, et al.W. Wohlleben, *Variation in dissolution behavior among different nanoforms and its implication for grouping approaches in inhalation toxicity.* NanoImpact, 2021. **23**(100341).

(3) Rasmsussen, K., J. Mast, P.J. De Temmerman, E. Verleysen, N. Waegeneers, F. Van Stern, and e. al., *Titanium dioxide, NM-100, NM-101, NM-102, NM-103, NM-104, NM-105: Characterization and physico-chemical properties.* . EUR 26637, 2014.

(4) Rasmussen, K., A. Mech, J. Mast, P.J. De Temmerman, N. Waegeneers, F. Van Steen, and e. al., *Synthetic amorphous silicon dioxide (NM-200, NM-201, NM-202, NM-203, NM-204): characterisation and physico-chemical properties.* JRC Scientific and Policy Reports, 2013.

(5) Singh, C., S. Friedrichs, M. Levin, R. Birkedal, K.A. Jensen, G. Pojana, and e. al., *NM-series of representative manufactured nanomaterials: Zinc oxide NM-110, NM-111, NM-112, NM-113 characterisation and test item preparation.* EUR 25066, 2011.

(6) Bove, P., M.A. Malvindi, S.S. Kote, R. Bertorelli, M. Summa, and S. Sabella, *Dissolution test for risk assessment of nanoparticles: a pilot study.* Nanoscale, 2017. **9**(19): p. 6315-6326.

(7) Carnovale, C., D. Guarnieri, L. Di Cristo, I. De Angelis, G. Veronesi, A. Scarpellini, and S. Sabella, *Biotransformation of Silver Nanoparticles into Oro-Gastrointestinal Tract by Integrated In Vitro Testing Assay: Generation of Exposure-Dependent Physical Descriptors for Nanomaterial Grouping.* Nanomaterials (Basel), 2021. **11**(6).

(8) 19057, I.T., *Nanotechnologies — Use and application of acellular in vitro tests and methodologies to assess nanomaterial biodurability.* 2017.
